# The HBx protein from hepatitis B virus coordinates a redox-active Fe-S cluster

**DOI:** 10.1016/j.jbc.2022.101698

**Published:** 2022-02-08

**Authors:** Chie Ueda, Michelle Langton, Jiahua Chen, Maria-Eirini Pandelia

**Affiliations:** Department of Biochemistry, Brandeis University, Waltham, Massachusetts, USA

**Keywords:** hepatitis B virus, iron–sulfur protein, iron, redox, scaffold protein, DDB1, DNA-damage binding protein 1, DsbC, disulfide bond isomerase C, EPR, electron paramagnetic resonance, HBV, hepatitis B virus, ROS, reactive oxygen species, Smc, structural maintenance of chromosomes, SSN, sequence similarity network

## Abstract

The viral protein HBx is the key regulatory factor of the hepatitis B virus (HBV) and the main etiology for HBV-associated liver diseases, such as cirrhosis and hepatocellular carcinoma. Historically, HBx has defied biochemical and structural characterization, deterring efforts to understand its molecular mechanisms. Here we show that soluble HBx fused to solubility tags copurifies with either a [2Fe-2S] or a [4Fe-4S] cluster, a feature that is shared among five HBV genotypes. We show that the O_2_-stable [2Fe-2S] cluster form converts to an O_2_-sensitive [4Fe-4S] state when reacted with chemical reductants, a transformation that is best described by a reductive coupling mechanism reminiscent of Fe-S cluster scaffold proteins. In addition, the Fe-S cluster conversions are partially reversible in successive reduction–oxidation cycles, with cluster loss mainly occurring during (re)oxidation. The considerably negative reduction potential of the [4Fe-4S]^2+/1+^ couple (−520 mV) suggests that electron transfer may not be likely in the cell. Collectively, our findings identify HBx as an Fe-S protein with striking similarities to Fe-S scaffold proteins both in cluster type and reductive transformation. An Fe-S cluster in HBx offers new insights into its previously unknown molecular properties and sets the stage for deciphering the roles of HBx-associated iron (mis)regulation and reactive oxygen species in the context of liver tumorigenesis.

Chronic hepatitis B virus (HBV) infection affects 300 million people globally and is a major risk factor for the development of hepatocellular carcinoma (1, 2). HBV is classified into 10 genotypes that appear in distinct geographical locations and are associated with different clinical outcomes or disease severity (3, 4). All HBV genotypes share a common genomic architecture featuring four overlapping genes (*P*, *S*, *C*, and *X*) encoding for seven viral proteins (5). Protein X (or HBx) is required for viral replication and is a key factor in chronic HBV-related hepatocarcinogenesis (6, 7, 8). HBx promotes transcription of the extrachromosomal HBV genome by hijacking the DNA-damage binding protein 1 (DDB1)-containing E3 ubiquitin ligase to target the “structural maintenance of chromosomes” (Smc) Smc5/6 complex for degradation (9). This mechanism stimulates transcription of episomal DNA templates as well as the HBV genome, giving HBx the title of a transcriptional transactivator (10). HBx has also been shown to promote viral replication by recruiting Parvulin 14 and 17 to the HBV covalently closed circular DNA to enhance transcriptional activation (11). In addition, HBx is reported to affect many oncogenic-related cellular processes, such as cell proliferation, DNA damage repair, and Fe regulation (12). For example, HBx has been linked to altered iron metabolism and iron regulatory protein 1 (IRP1) expression in hepatic cells, and is suggested to inhibit the tumor suppressor PTEN via the production of reactive oxygen species (ROS) (13, 14). Most of our current understanding of HBx is inferred from cellular experiments (reviewed in detail in the following refs: (12, 15, 16)), and thus the molecular mechanisms that underlie HBx activity remain obscure.

HBx is a small 154 amino acid protein with a cysteine-rich sequence that is highly conserved across the various HBV genotypes. Naturally occurring C-terminally truncated (30–40 aa) or N-terminally elongated (52 aa) forms of HBx are associated with different disease outcomes (17, 18). Although both canonical and noncanonical HBx proteins share pronounced sequence identity, the lack of sequence similarity to any known proteins hinders inference of molecular structure and function. The historically poor solution behavior of HBx has confined *in vitro* studies to samples that are nonnative, highly truncated, or contain extensive amino acid substitutions, often leading to conflicting outcomes and inadequate biophysical and biochemical characterization (19, 20, 21). Solubility enhancement tags have successfully provided a first insight into the molecular properties of HBx, especially in its metal binding potential. HBx fused to the Maltose Binding Protein (MBP-HBx) copurifies with significant amounts of Fe and Zn (20) and was recently assigned as a Zn-binding protein based on the copurification of Zn when coexpressed or fused to DDB1 (22). C61, C69, C137, and H139 were identified as potential ligands to the Zn cofactor, and the residues of this CCCH motif were shown to be critical for HBx viral replication function (*i.e.*, targeting the Smc5/6 complex for degradation). However, there was no evidence that Zn binding facilitates protein–protein interactions with DDB1, leaving the functional role of Zn binding in HBx unresolved.

In the present study, we focus on a soluble construct of HBx fused to disulfide bond isomerase C (DsbC-HBx) (23) to delve into the potential of HBx to coordinate transition metal ions. We find that HBx copurifies with Fe assembled into an Fe-S cluster, establishing HBx as an unrecognized Fe-S protein. HBx binds two different types of Fe-S clusters: a [2Fe-2S] or [4Fe-4S] cluster depending on the presence or absence of oxygen, respectively. Through multiple spectroscopic approaches, we characterize the chemical nature and redox attributes of the newfound Fe-S cluster in HBx. Interestingly, the [2Fe-2S] cluster form undergoes a reductive rearrangement that affords formation of [4Fe-4S] clusters. This transformation is best described by a reductive coupling mechanism that is a well-studied and characteristic feature of Fe-S cluster scaffold proteins, such as IscU and IscA. This parallel is intriguing and invokes the notion that HBx may potentially hijack cellular pathways by affecting Fe-S cluster biogenesis (24). Despite this similarity, however, there are multiple ways by which the Fe-S cluster in HBx may be important either at a structural or a regulatory level. Fe-S clusters are functionally diverse and ubiquitous cofactors that are becoming increasingly recognized as key components in viral proteins from rotaviruses, polyomaviruses, and more recently coronaviruses (25, 26, 27, 28, 29). The roles of Fe-S cofactors in viral proteins are proposed to be structural with critical outcomes in the host protein activity. In our study, the presence of the newfound Fe-S cluster sets the stage for interrogating the molecular and structural underpinnings of HBx, whereas its redox-dependent behavior outlines possible roles of the Fe-S cluster-bound HBx in Fe homeostasis.

## Results

### HBx is an Fe-S cluster–binding protein

**Figure 1:**
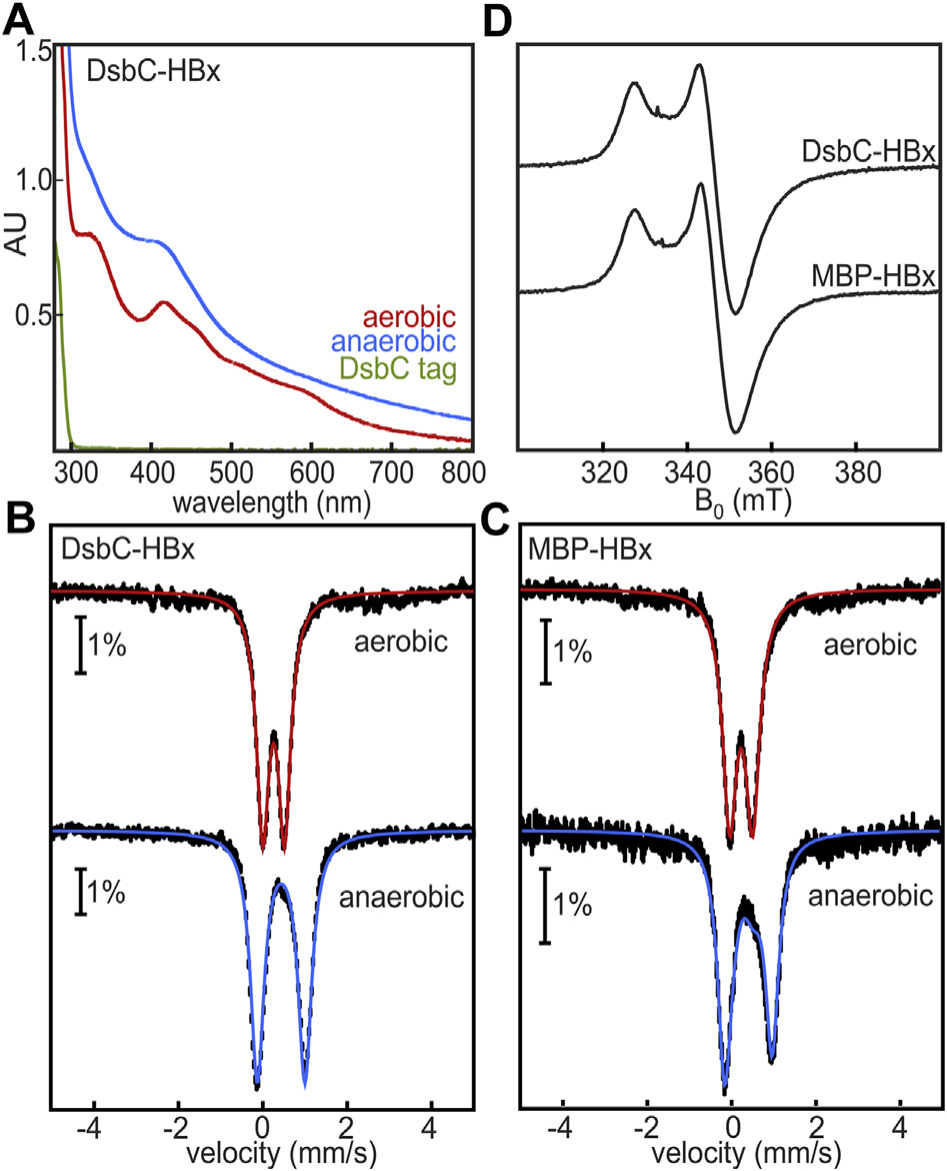
**HBx is an Fe-S-binding protein.***A*, UV-visible spectra of DsbC-HBx isolated aerobically (*red line*) and anaerobically (*blue line*). DsbC (*green line*) shows no spectral features. *B,* Mössbauer spectra of DsbC-HBx aerobically (*top*) and anaerobically (*bottom*) isolated. *C*, Mössbauer spectra of MBP-HBx aerobically (*top*) and anaerobically (*bottom*) isolated. Mössbauer spectra were recorded at 4.2 K with a magnetic field (B = 78 mT) applied parallel to the γ-beam. *D*, X-band continuous wave (cw) EPR spectra of dithionite-reduced DsbC-HBx and MBP-HBx (microwave frequency = 9.38 GHz, T = 10 K, microwave power = 0.64 mW, modulation amplitude = 1 mT).

Aerobically isolated DsbC- or MBP-HBx copurifies with 2.4 to 2.5 mol Fe per mol protein (Table S1). Both proteins exhibit a reddish brown color and optical features at 325, 415, and 460 nm, suggestive of the presence of a [2Fe-2S] cluster (30) (Fig. 1*A*). ^57^Fe-enriched samples were examined by Mössbauer spectroscopy to confirm the exact chemical nature of the bound Fe species. The 4.2 K Mössbauer spectrum of the aerobically isolated DsbC-HBx was collected in a small magnetic field (78 mT) applied parallel to the direction of the γ irradiation (Fig. 1*B*) and shows a quadrupole doublet with parameters characteristic of antiferromagnetically coupled all-ferric [2Fe-2S]^2+^ clusters (δ = 0.28 mm/s, ΔE_Q_ = 0.51 mm/s, ∼84% of the total Fe absorption) (Table S2) (31, 32). A broad baseline spectrum makes up the rest of the signal intensity (16%) and is attributed to adventitiously bound high-spin (*S* = 5/2) Fe^3+^. The Mössbauer spectrum of the aerobically isolated MBP-HBx is essentially identical to that of DsbC-HBx (Fig. 1*C*). Elemental analyses in tandem with Mössbauer spectroscopy indicate binding of one [2Fe-2S] cluster per polypeptide. Exact Fe-S cluster stoichiometry cannot be unequivocally determined because both fusion constructs purify as poorly defined higher-order oligomers as previously reported (20). The observed aggregation is partially due to intermolecular disulfides (Fig. S1) and unknown interactions that are salt and concentration independent.

The HBx fusion proteins were also isolated under O_2_-free conditions to examine whether the [2Fe-2S] cluster arises from oxidative degradation of a more O_2_-sensitive [4Fe-4S] cluster form. The UV-visible spectrum of the anaerobically purified DsbC-HBx exhibits a band at 420 nm, consistent with the presence of [4Fe-4S] clusters (Fig. 1*A*) (30). The 4.2 K/78 mT Mössbauer spectrum of the anaerobically purified DsbC-HBx is dominated by a single quadrupole doublet with δ = 0.45 mm/s and ΔE_Q_ = 1.11 mm/s (Fig. 1*B*), confirming the presence of [4Fe-4S]^2+^ clusters and supporting the hypothesis that the [2Fe-2S] clusters detected in the aerobically isolated protein are oxidative products. The anaerobically purified MBP-HBx has a similar spectrum with the [4Fe-4S]^2+^ cluster as the major component (60% of total Fe absorption). The remaining absorption can be ascribed to a subspectrum of [2Fe-2S]^2+^ clusters (∼17%), and a broad paramagnetic subspectrum assigned to Fe polysulfides formed during the purification process (∼23%) (Fig. 1*C*). Parallel experiments on whole *Escherichia* *(E.)* *coli* cells monitored by Mössbauer spectroscopy showed that the [2Fe-2S] cluster form is also present in whole cells (Fig. S2), suggesting that [2Fe-2S] clusters in HBx may be both an oxidative outcome and a consequence of incomplete Fe-S cluster insertion due to overexpression in *E. coli*. There is no evidence for formation of [3Fe-4S]^1+^ clusters in the aerobically purified protein, as demonstrated by electron paramagnetic resonance (EPR) (Fig. S3); therefore, the [2Fe-2S]^2+^ form appears to be the only stable oxidative by-product of the [4Fe-4S] cluster form.

Dithionite reduction of the aerobically isolated DsbC- and MBP-HBx yields EPR spectra with identical parameters and lineshape irrespective of the solubility tag (Fig. 1*D*). The observed signals have axial symmetry, principal g-values of 2.04, 1.94, and 1.94, and relaxation properties that are best attributed to *S* = 1/2 [4Fe-4S]^1+^ clusters rather than [2Fe-2S]^1+^ clusters (Figs. 1*D* and S4). These data suggest that reductive treatment generates [4Fe-4S]^1+^ clusters, possibly as a result of reductive coupling (*vide infra*). This process, which has been observed in Fe-S cluster chaperone proteins and proteins that act as Fe-S cluster donors, involves the one-electron reduction of [2Fe-2S]^2+^ to [2Fe-2S]^1+^ clusters, followed by the coupling of two [2Fe-2S]^1+^ clusters to form one [4Fe-4S]^2+^ cluster. Cleavage of the DsbC tag by tobacco etch virus protease does not result in Fe-S cluster loss (as seen by EPR, Fig. S5); however, we were unable to separate out the DsbC tag.

### Fe-S cluster binding is a common feature of HBx across different genotypes

**Figure 2:**
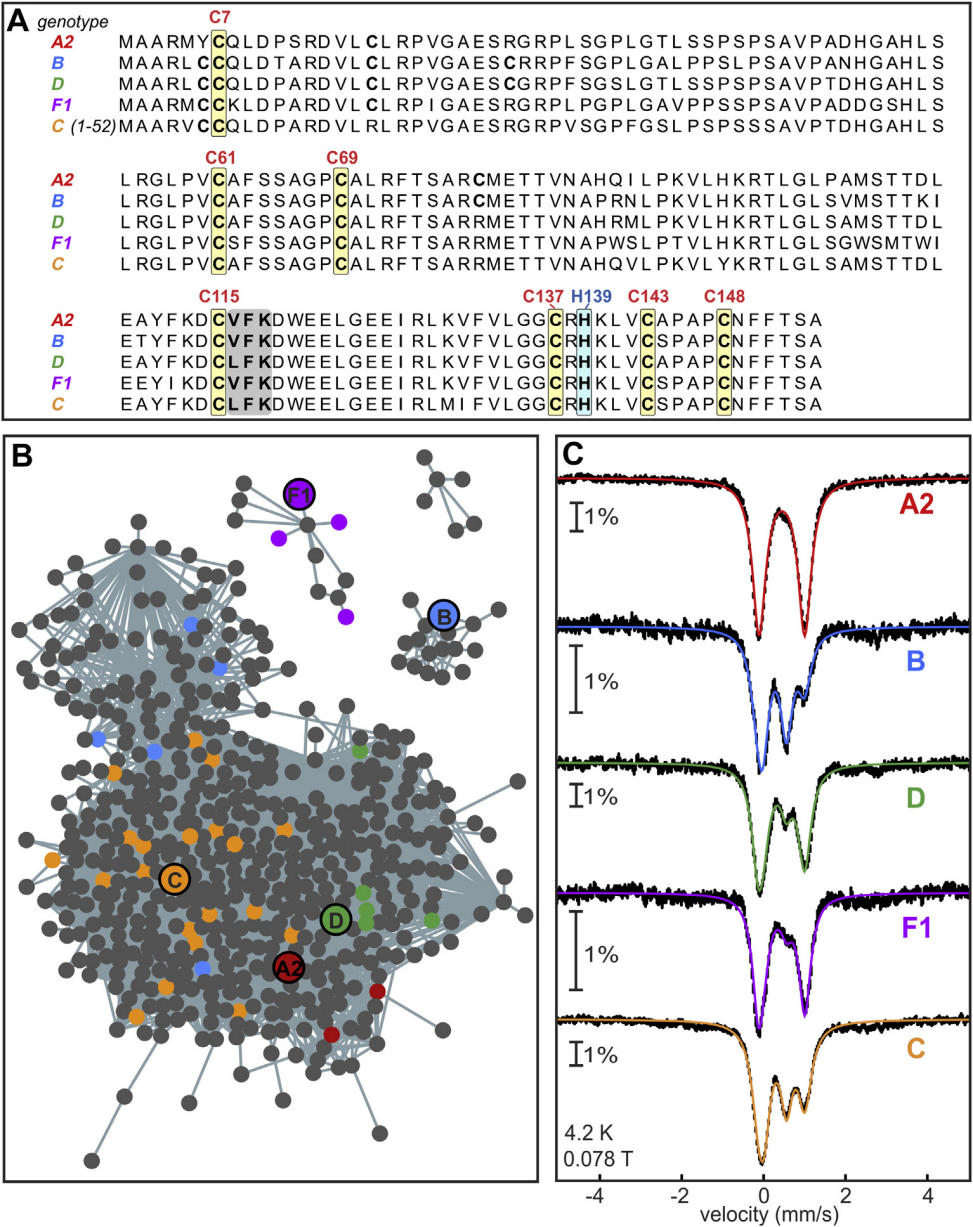
**Sequence alignment, Sequence Similarity Network (SSN), and Mössbauer spectra of HBxfrom select HBV genotypes.***A*, sequence alignment of HBx from HBV genotypes A2, B, C, D, and F1. The N-terminal 52 amino acids of HBx from genotype C have been omitted. The seven strictly conserved cysteines and conserved histidine are highlighted in *yellow* and *blue*, respectively. The LYR-like motif (VFK) is shaded in a *gray box*. *B*, SSN of HBx proteins colored according to their HBV genotype. Nodes (*circles*) include sequences of HBx with ≥95% sequence identity. Edges connect nodes having ≥90.6% sequence identity. *Bold circles* represent the sequences used in this study. Clusters with <4 nodes (240 clusters) were not included in the figure. *C*, Mössbauer spectra of DsbC-HBx from genotypes A2, B, C, D, and F1 isolated anaerobically. Spectra were recorded at 4.2 K with magnetic field (B = 78 mT) applied parallel to the γ-beam. The fits represent the total simulations of the Fe-S content in the sample.

Bioinformatic analyses identified ∼6800 HBx sequences that share a high degree of sequence identity (>80%). HBx sequences are rich in cysteines, seven of which are invariant (Fig. 2*A*). Because Fe-S cluster binding is primarily cysteine dependent, we suspect that some of these conserved cysteines are involved in cofactor binding in HBx. Other residues, such as histidine, aspartate, and arginine can also contribute to Fe-S cluster coordination (33). In this respect, the strictly conserved H139 may also serve as a ligand. Single substitution of each of the conserved cysteines (Fig. 2*A*) with alanine did not affect Fe-S incorporation, and only a variant lacking all cysteines was unable to coordinate an Fe-S cluster (Fig. S6). We also identified a highly conserved leucine–tyrosine–arginine (LYR)-like motif in the HBx primary sequence (Fig. 2*A*). Such a motif has been shown to be a recognition sequence for direct interaction with the Fe-S cochaperone HSC20 (34), which may further support the fact that HBx can receive an Fe-S cluster from the biosynthetic machinery.

Although the sequence similarity network (SSN) maps little segregation due to high sequence identity, it is overall clustered by the extant HBV genotype. HBx from the HBV genotypes A, C, and D (shown in red, orange, and green, respectively, Fig. 2*B*) exhibit the highest sequence homology to each other. Genotypes A and D are distributed pandemically in populations worldwide, whereas genotype C is found primarily in East Asia (3, 4). From genotype C we selected a sequence designated “whole-HBx” or wHBx, which includes an N-terminal 52-aa extension, to increase sequence variability. The two most sequence-diverse genotypes that cluster separately in the SSN (*i.e.*, B and F1) are mainly found in East Asia and South America, respectively (3, 4). On the basis of their distribution and clinical importance, DsbC fusions were generated with select sequences from the A2, B, C, D, and F1 HBV genotypes. All five proteins were ^57^Fe-enriched for characterization by Mössbauer spectroscopy and isolated under O_2_-free conditions. The isolated DsbC-HBx fusions all coordinate both [2Fe-2S]^2+^ and [4Fe-4S]^2+^ clusters (Fig. 2*C*), albeit at different relative ratios (Table S3), demonstrating that Fe-S cluster binding is a common feature across diverse HBx sequences.

### Zn may substitute for the Fe-S cluster in HBx

**Figure 3:**
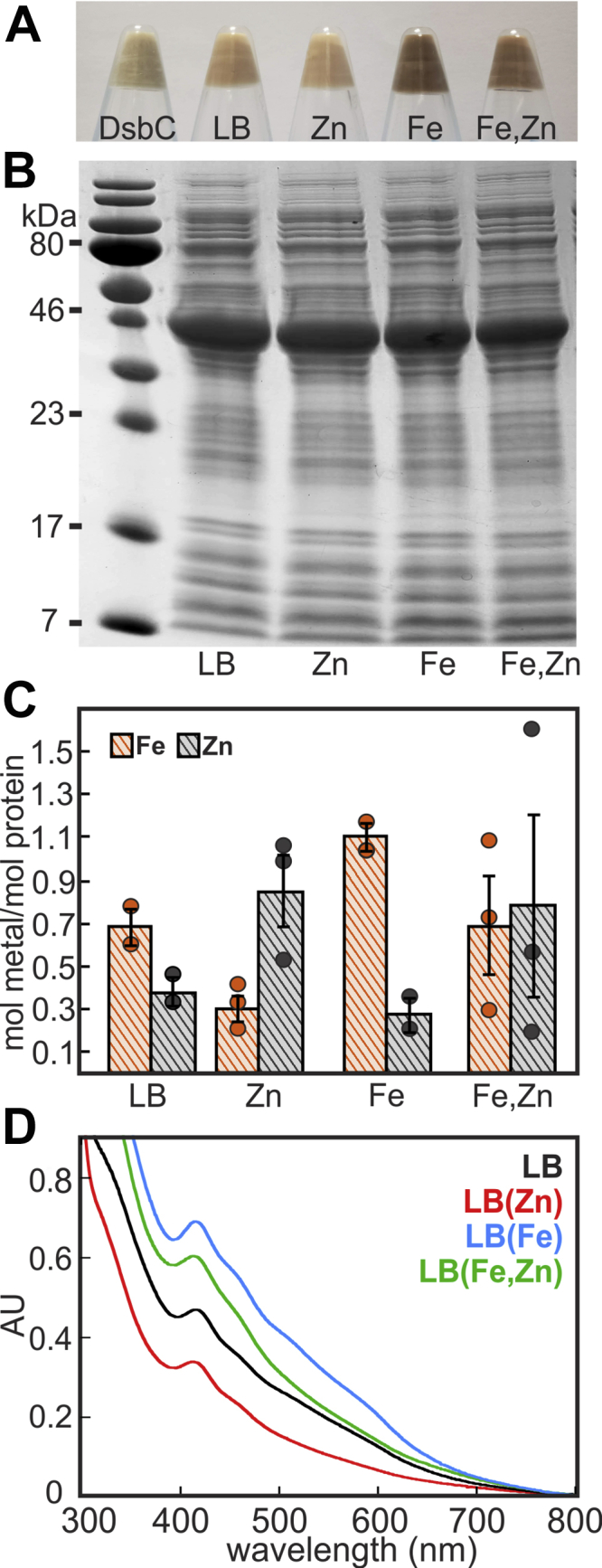
**Metal binding properties of DsbC-HBx.***A*, *E. coli* cell pellets of DsbC-HBx expressed with different metal supplementation, from *left* to *right*: DsbC tag in LB, LB only, LB + 250 μM ZnSO_4_, LB + 250 μM Fe(NH_4_)_2_(SO_4_)_2_, LB + 250 μM each ZnSO_4_ and Fe(NH_4_)_2_(SO_4_)_2_. *B*, SDS-PAGE gel of normalized cell pellets, from *left to right*: LB only, LB + 250 μM ZnSO_4_, LB + 250 μM Fe(NH_4_)_2_(SO_4_)_2_, LB + 250 μM each ZnSO_4_ and Fe(NH_4_)_2_(SO_4_)_2_. *C*, inductively coupled atomic emission spectrometry analysis of metal content of purified DsbC-HBx expressed under different metal supplementation conditions. *Circles* represent individual data points; error bars represent the standard error of the mean. *D*, UV-visible spectra of purified DsbC-HBx expressed under different metal supplementation conditions.

DsbC-HBx was expressed in LB media supplemented with Fe and Zn salts to examine the extent of cofactor incorporation on the basis of metal availability. Cells overexpressing DsbC-HBx exhibit a light brown color, indicating enrichment in Fe-S clusters, whereas cells overexpressing DsbC alone lack color (Fig. 3*A*). Therefore, cell pellet color is an initial indicator for Fe-S cluster incorporation under different metal supplementation conditions. Supplementation with 250 μM ZnSO_4_ causes a lighter-colored pellet, whereas, supplementation with 250 μM Fe(NH_4_)_2_(SO_4_)_2_ results in a dark brown pellet (Fig. 3*A*). DsbC-HBx expression with simultaneous Zn and Fe supplementation leads to brown cell pellets that are lighter colored than those obtained from cells supplemented with Fe alone, suggesting that Zn may partially inhibit Fe incorporation when added in equimolar amounts. SDS-PAGE analysis of normalized cell pellets shows a similar expression level of the ∼42-kDa DsbC-HBx in the LB, LB(Fe), LB(Zn), and LB(Fe,Zn) cells (Fig. 3*B*). The optical spectra of the aerobically purified proteins show formation of a [2Fe-2S]^2+^ cluster under all conditions, but signal intensity is decreased when Zn is added to the media, consistent with compromised Fe-S incorporation (Fig. 3, *C* and *D*). These results highlight that (i) Zn competes with the Fe-S cluster for binding to HBx, suggestive of a common coordination site, and (ii) Fe-S cofactor assembly is favored over Zn incorporation supporting that the Fe-S cluster may be the preferred cofactor *in cellulo*.

### The [4Fe-4S] cluster in HBx may be formed by reductive coupling of two [2Fe-2S] clusters

#### Stoichiometric reduction

**Figure 4:**
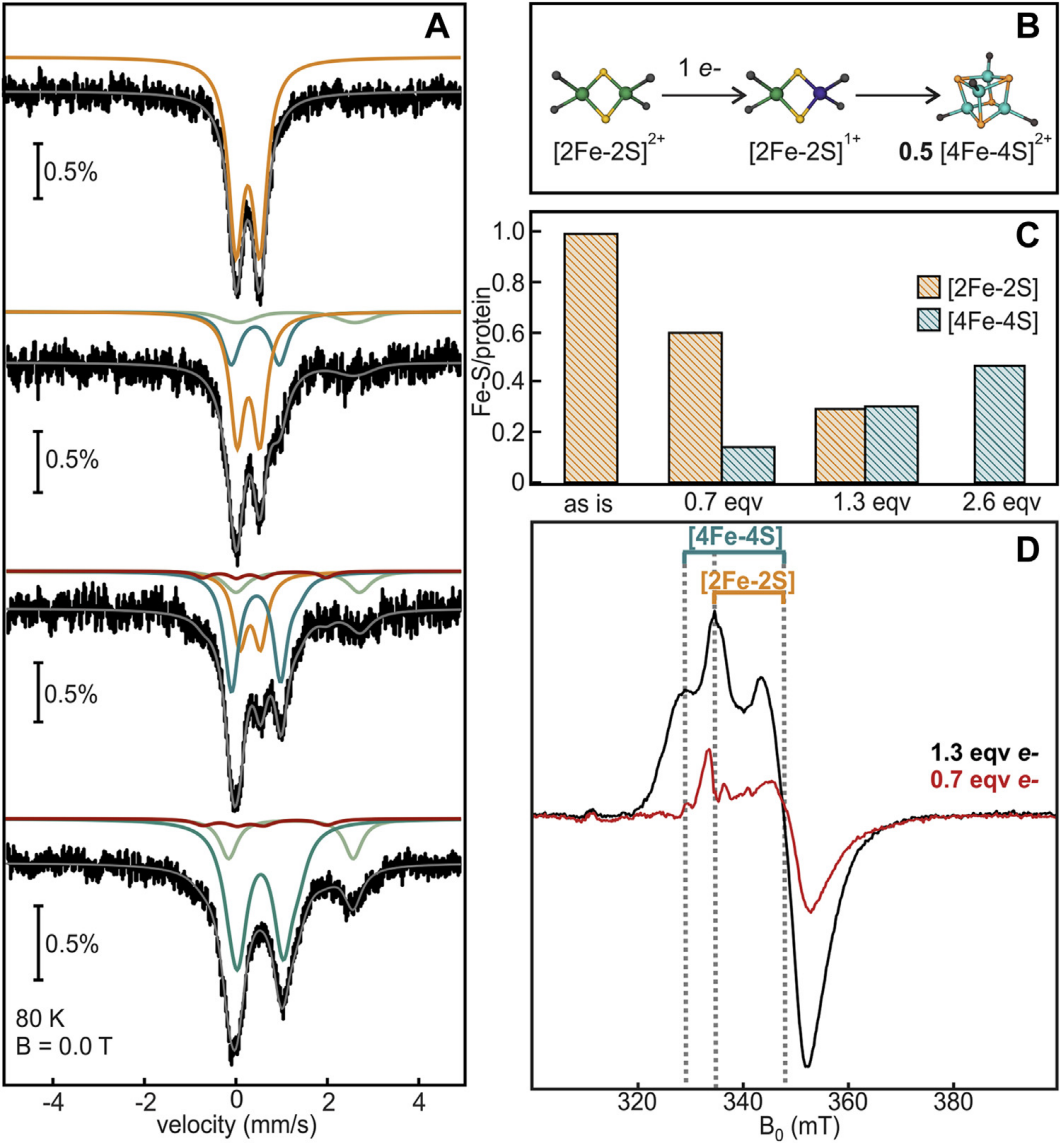
**Aerobically isolated DsbC-HBx reacted with stoichiometric reducing equiv.***A*, Mössbauer spectra recorded at 80 K in the absence of an external magnetic field. Spectra from *top* to *bottom*: aerobically isolated, 0.7 reducing equiv, 1.3 reducing equiv, 2.6 reducing equiv. The fits of quadrupole doublet subspectra are shown in *solid lines*: *orange*, [2Fe-2S]^2+^ clusters; *red*, [2Fe-2S]^1+^ clusters; *teal*, [4Fe-4S]^1+/2+^ clusters; *light green*, Fe^2+^. *B*, scheme of reductive coupling. *C*, graphical representation of the speciation of Fe-S clusters using relative ratios determined from the 80 K Mössbauer spectral fits. *D*, X-band cw EPR spectra of parallel stoichiometric reductions of DsbC-HBx (microwave frequency = 9.38 GHz, T = 10 K, microwave power = 0.64 mW, modulation amplitude = 1 mT). *Gray dotted lines* denote the position of the principal g-values of the Fe-S signals.

The aerobically purified DsbC-HBx coordinates 1.0 [2Fe-2S] cluster per protein (Figs. 4 and S7) and some adventitiously bound high-spin Fe^3+^, which is inaccessible to chelating agents. The observed formation of [4Fe-4S]^1+^ clusters by dithionite reduction of DsbC-HBx:[2Fe-2S] in EPR (Fig. 1*D*) prompted us to examine this process in more detail by ^57^Fe Mössbauer spectroscopy. The [2Fe-2S] to [4Fe-4S] cluster transformation requires a strong reducing agent (*i.e.*, sodium dithionite) and is not induced by weaker thiol-based reductants such as DTT or by the *Azotobacter (A.) vinelandii isc*Fdx (Fig. S8). This implies that the reduction potential of DsbC-HBx:[2Fe-2S] is fairly negative when compared with canonical [2Fe-2S] clusters (30, 33). In a first experiment we mimicked the conditions employed previously for the bacterial IscU, in which reductive transformation of [2Fe-2S] clusters was afforded by stoichiometric addition of reducing equiv (24). Because the [4Fe-4S] cluster in DsbC-HBx can be reduced to the 1+ state, in contrast to that of IscU that reductively degrades, Mössbauer spectra were recorded at 4.2 and 80 K because spin relaxation for paramagnetic species is temperature dependent (31, 32) (Figs. 4*A* and S7). Upon reaction with 0.7 reducing equiv per protein, DsbC-HBx:[2Fe-2S] exhibits a spectrum that demonstrates loss of 0.35 [2Fe-2S]^2+^ clusters and formation of 0.15 [4Fe-4S]^2+^ clusters per DsbC-HBx (Fig. 4*C*). The nonstoichiometric conversion may be rationalized by loss of reducing equiv toward the one-electron reduction of adventitiously bound Fe^3+^ and [2Fe-2S]^2+^ clusters (the latter verified by EPR by appearance of the axial signal with principal values g_II_ = 2.00, g_⊥_=1.93) (Fig. 4*D*). Addition of 1.3 reducing equiv leads to an overall loss of 0.65 [2Fe-2S]^2+^ clusters and formation of 0.3 [4Fe-4S]^2+/1+^ clusters per DsbC-HBx. We do not attain the expected 0.35 [4Fe-4S] clusters due to reduction of adventitious Fe^3+^ and further reduction of [4Fe-4S]^2+^ to [4Fe-4S]^1+^ clusters. Reduction with 2.6 reducing equiv affords complete loss of the parent [2Fe-2S]^2+^ centers and formation of 0.5 [4Fe-4S] clusters per DsbC-HBx, 0.4 of which are in the 2+ state, whereas the rest are in the reduced 1+ state. In the whole reductive process, the amount of Fe^2+^ (δ = 1.27 mm/s and ΔE_Q_ = 2.67 mm/s) formed corresponds to the reduction of the initial Fe^3+^ present in the sample, demonstrating that reductive degradation of Fe-S clusters is negligible (Table S4). Despite the unavoidable consumption of electrons by adventitious free Fe and further reduction of the nascent [4Fe-4S]^2+^ clusters (Fig. 4*D*), our findings are consistent with a reductive coupling mechanism like that observed for IscU.

#### Time-dependent reduction

**Figure 5:**
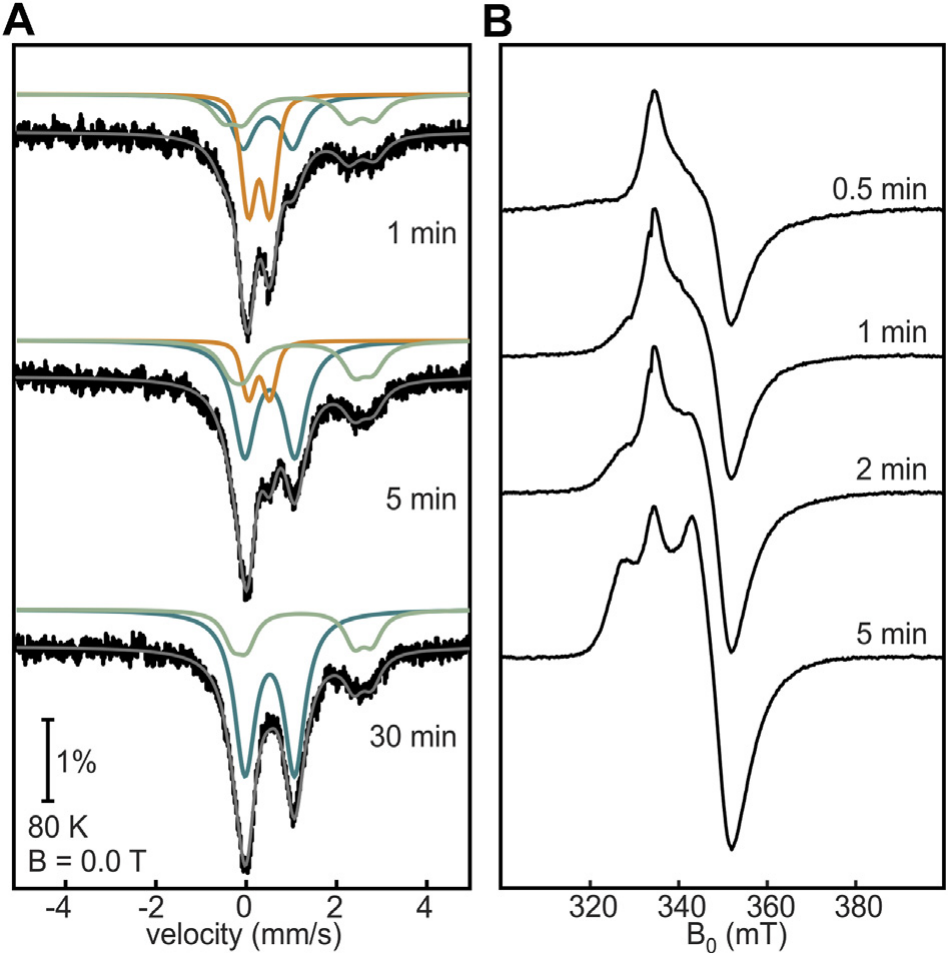
**Time-dependent reduction of aerobically isolated DsbC-HBx.***A*, Mössbauer spectra were recorded at 80 K in the absence of an external magnetic field. Total simulations are shown as *gray lines* and are the addition of the subspectra (*orange*, [2Fe-2S]; *teal*, [4Fe-4S]; *light green*, Fe^2+^). *B*, X-band cw EPR spectra of DsbC-HBx (microwave frequency = 9.38 GHz, T = 10 K, microwave power = 0.64 mW, modulation amplitude = 1 mT).

The time-dependent reduction of the [2Fe-2S] to [4Fe-4S] cluster conversion was also monitored by Mössbauer and EPR spectroscopies in an analogous experiment to the one performed for the ^Nif^IscA chaperone (35). The starting sample contained 0.8 [2Fe-2S] clusters per protein (Fig. S9) and was reacted with excess sodium dithionite for 1, 5, and 30 min prior to freezing (Fig. 5*A*). Already after 1 min, there is a loss of 0.4 [2Fe-2S]^2+^ clusters and formation of 0.2 [4Fe-4S] clusters, while at 5 min 0.65 [2Fe-2S]^2+^ clusters have been reductively consumed and 0.3 [4Fe-4S]^2+/1+^ clusters have been formed. At 30 min, all [2Fe-2S]^2+^ clusters initially present in the sample are reduced, yielding 0.4 [4Fe-4S]^2+/1+^ clusters as a result of reductive coupling and Fe^2+^ stemming mostly from the reduction of adventitious Fe^3+^ in the aerobically isolated sample (Table S5). In parallel EPR time-dependent reduction experiments, the [2Fe-2S]^1+^ state is observed as early as 0.5 min into reduction and maximizes at 5 min, after which the signal decays and completely disappears by 30 min, in agreement with the Mössbauer experiments (Figs. 5*B* and S9). At 5 min the [4Fe-4S]^1+^ state is detectable, indicating that electrons are concurrently used for reductive coupling and the further reduction of the newly formed [4Fe-4S]^2+^. Collectively, these results are consistent with a reductive coupling scheme in which the [2Fe-2S]^1+^ cluster forms as an intermediate in the production of [4Fe-4S] clusters.

### Midpoint potential of the [4Fe-4S]^2+/1+^ redox couple

**Figure 6:**
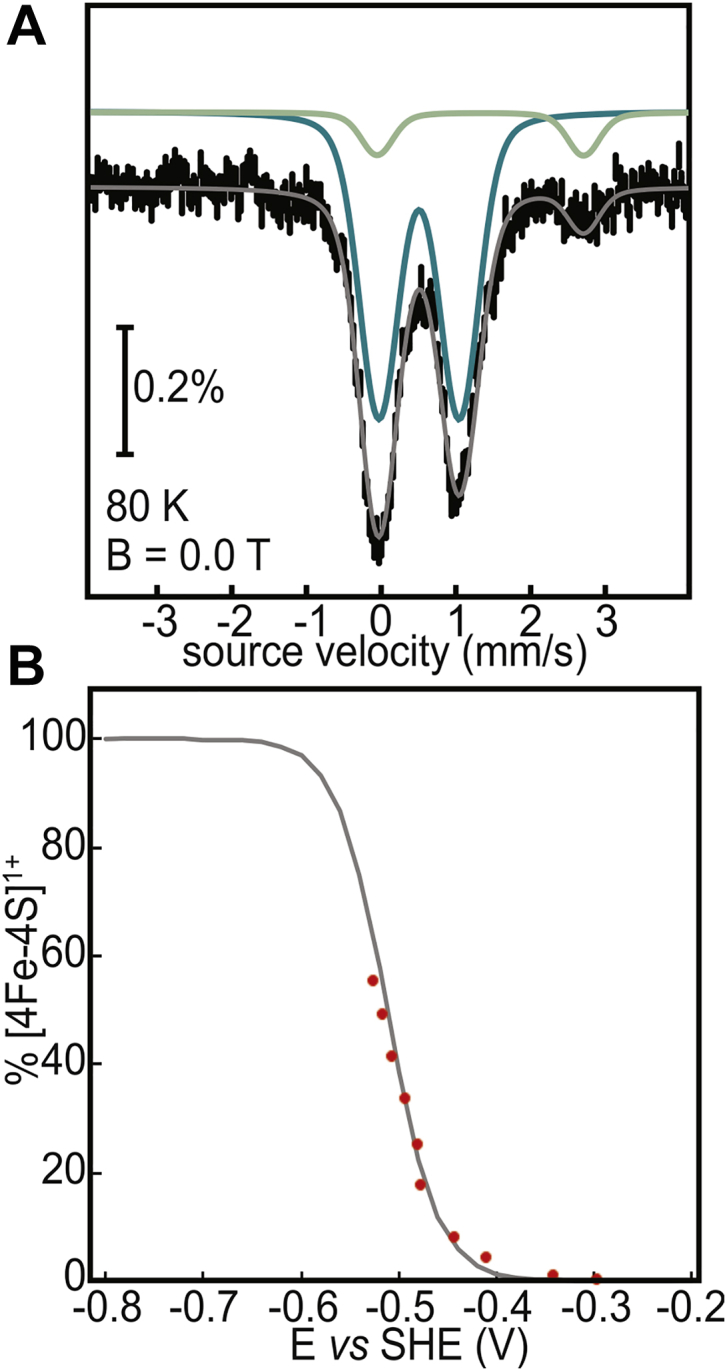
**Reduction of Dsbc-HBx:[4Fe-4S].***A*, Mössbauer spectrum of anaerobically isolated DsbC-HBx reduced with an excess of sodium dithionite (10 mM) for 30 min recorded at 80 K and in the absence of an external magnetic field. The total simulation (*gray line*) is a sum of two subspectra (*teal*, [4Fe-4S]; *light green*, Fe^2+^). *B*, redox titration of DsbC-HBx as a function of potential at pH 8.0. The *gray line* corresponds to a fit considering a one-electron transition and was calculated using the Nernst equation.

The 80 K Mössbauer spectrum of the dithionite reduced DsbC-HBx:[4Fe-4S] consists of a major component (90% of the Fe absorption) attributed to both oxidized and reduced [4Fe-4S] clusters and a minor component (10% of the Fe absorption) assigned to high-spin Fe^2+^ (Fig. 6*A*). Treatment of the [4Fe-4S]^2+^ state with excess dithionite, therefore, does not lead to significant cluster disassembly, unlike the IscU chaperone for which complete cluster decomposition is observed (36). The lack of reductive degradation of the [4Fe-4S] cluster enabled us to measure the midpoint potential of the [4Fe-4S]^2+/1+^ couple by EPR potentiometric titrations on a [4Fe-4S] cluster-enriched sample (Fig. S10). The fraction of [4Fe-4S]^1+^ clusters was followed as a function of the measured potential (Fig. 6*B*). At the lowest attainable potential (−525 mV), only 55% of the [4Fe-4S]^2+^ clusters present in the sample were reduced to the 1+ state, precluding measurement of the complete titration curve. The estimated midpoint potential (E_m_) for the [4Fe-4S]^2+/1+^ couple in HBx is −520 mV (Fig. 6*B*).

### Reversibility of the [2Fe-2S] to [4Fe-4S] cluster conversion

**Figure 7:**
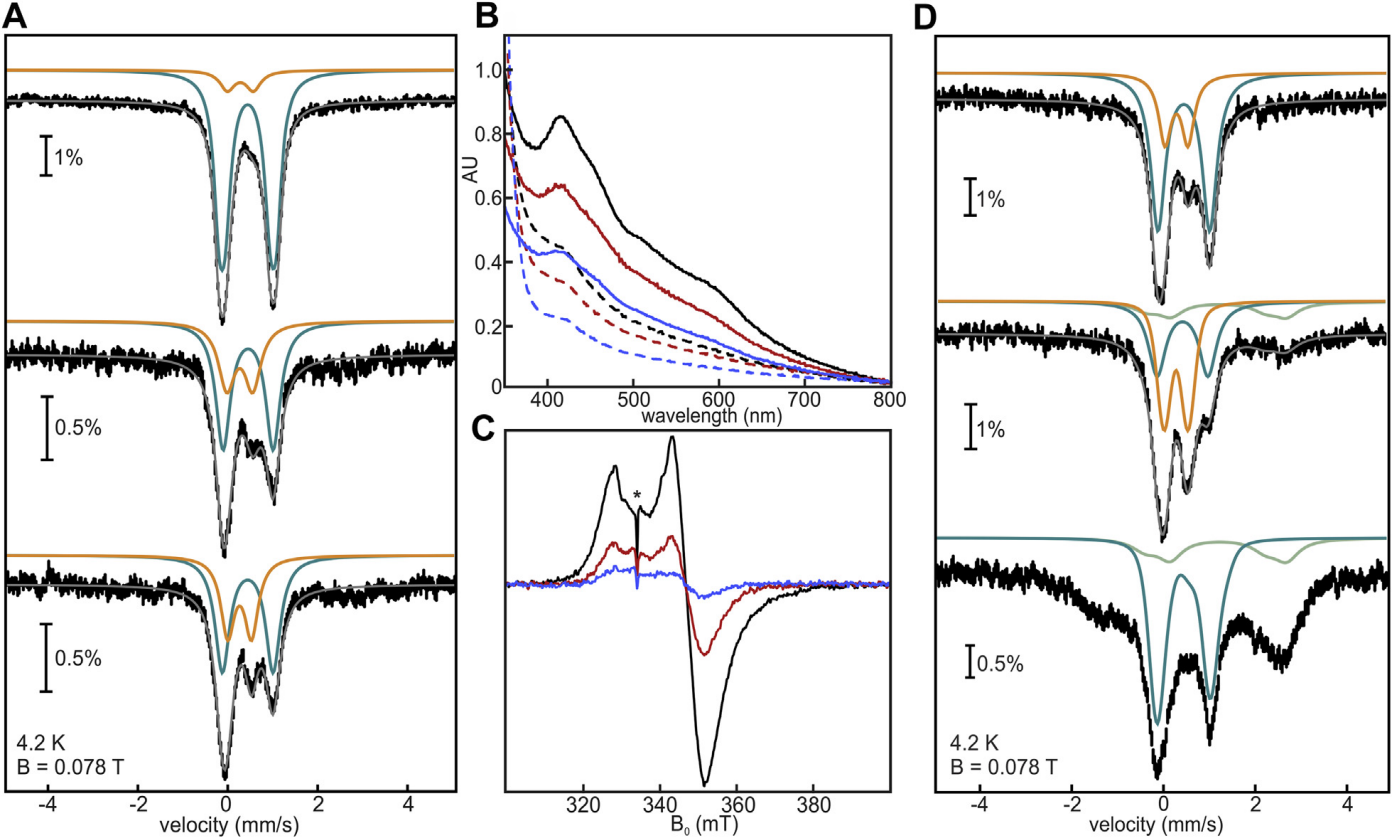
**Oxygen exposure and redox cycling of DsbC-HBx.** All Mössbauer spectra were recorded at 4.2 K in the presence of a magnetic field (B = 78 mT) applied parallel to the γ-beam. The total simulation (*gray lines*) for all experimental Mössbauer spectra (*vertical bars*) are sums of the fits of the subspectra (*orange*, [2Fe-2S]; *teal*, [4Fe-4S]; *light green*, Fe^2+^). *A*, *top*, anaerobically isolated DsbC-HBx, (*middle*) exposed to 1 equiv of O_2_, (*bottom*) exposed to 2 equiv of O_2_. *B*, redox cycling monitored by UV-visible spectroscopy. The initial sample (*black solid line*) was reduced with dithionite (*dashed black line*), exposed to air for 1 h (*solid orange line*), reduced again with dithionite (*dashed orange line*), exposed to air for 1 h (*solid blue line*), and finally reduced again with dithionite (*dashed blue line*). *C*, redox cycling monitored by X-band cw EPR spectroscopy (microwave frequency = 9.38 GHz, T = 10 K, microwave power = 0.64 mW, modulation amplitude = 1 mT). The experiment analogous to (*B*) was performed: one reduction (*black*), two rounds of reduction (*orange*), three rounds of reduction (*blue*). ∗A small sharp signal present in all samples is not Fe-S cluster related and arises from subtraction of a paramagnetic impurity in the resonator. *D*, *top*, anaerobically isolated DsbC-HBx, (*middle*) exposed to air for 4 h, (*bottom*) rereduced with excess sodium dithionite.

We exposed DsbC-HBx:[4Fe-4S] to molecular O_2_ to monitor the extent of [2Fe-2S] cluster formation and other oxidative products by Mössbauer spectroscopy (Fig. 7*A*). A sample containing 0.5 [4Fe-4S] and 0.1 [2Fe-2S] clusters per protein was first reacted with 1 equiv of O_2_ per [4Fe-4S] cluster. The resulting spectrum shows a loss of 0.2 [4Fe-4S] clusters and formation of 0.23 [2Fe-2S] clusters. The rest of the spectral intensity consists of broad paramagnetic signals of high-spin Fe^3+^ stemming from oxidative disassembly of 0.1 [4Fe-4S] clusters. Upon addition of 2 equiv of O_2_, there is cumulative loss of 0.26 [4Fe-4S] clusters, gain of 0.3 [2Fe-2S] clusters, and degradation of 0.10 [4Fe-4S] to Fe^3+^. Oxidative conversion from [4Fe-4S] to [2Fe-2S] clusters is therefore only ∼60% efficient, with a significant fraction of the Fe-S clusters oxidatively degrading to Fe^3+^ (Table S6). The possibility of the small amount of paramagnetic species observed to be assigned to [3Fe-4S]^1+^ clusters cannot be excluded; however, the resonances are too broad to be consistent with formation of a trinuclear cluster, and also, we have no evidence for accumulation of [3Fe-4S]^1+^ clusters in the aerobically exposed DsbC-HBx (Figs. 1*B*, S3, and S7). Therefore, we consider the formation of [3Fe-4S]^1+^ clusters unlikely.

To examine the extent of reversibility of the [2Fe-2S] to [4Fe-4S] transformation, we subjected DsbC-HBx:[2Fe-2S] to consecutive reduction–oxidation cycles while monitoring Fe-S cluster species by UV-visible and EPR spectroscopy. Dithionite reduction of the as-purified DsbC-HBx leads to the disappearance of the [2Fe-2S]^2+^ cluster optical features (*i.e.*, 415 and 460 nm) and appearance of a single band at 420 nm, consistent with the formation of [4Fe-4S] clusters (Fig. 7*B*). After removing the reductant, the air-exposed sample recovers the [2Fe-2S]^2+^ cluster optical spectrum, but to a lower extent. The optical features progressively bleach upon multiple rounds of oxidation and reduction, but cycle between the [2Fe-2S] and [4Fe-4S] cluster forms, showing that they may interconvert multiple times. Cognate EPR experiments were conducted using the [4Fe-4S]^1+^ state as a quantitative probe of the Fe-S clusters remaining in the sample after each reduction step (Fig. 7*C*). If the first reduction corresponds to the maximal attainable [4Fe-4S]^1+^ clusters, the signal is reduced to 32% and 11% after the second and third reduction steps, respectively. Overall, these results show that the oxidatively formed [2Fe-2S] clusters can undergo successive rounds of reductive coupling; however, there is significant Fe-S cluster degradation upon each reduction–oxidation cycle (Table S6).

To precisely determine the fate of the Fe-S species during reduction–oxidation, analogous experiments were performed by Mössbauer spectroscopy (Fig. 7*D*). The initial DsbC-HBx:[4Fe-4S] sample consists of 0.3 [4Fe-4S]^2+^ clusters and 0.25 [2Fe-2S]^2+^ clusters. This sample was exposed to air for 4 h in the Mössbauer cup and refrozen. The resultant spectrum shows oxidative loss of 0.15 [4Fe-4S] clusters accompanied by an increase of 0.1 [2Fe-2S] clusters. The rest of the Fe appears in mononuclear Fe^2+^ and Fe^3+^. Rereduction of the oxygen-exposed sample with sodium dithionite results in a spectrum containing 0.4 [4Fe-4S] clusters, demonstrating the reductive coupling of the oxidatively formed [2Fe-2S] clusters. The remaining Fe in the sample (20% of the overall Fe absorption) is assigned to mononuclear Fe^2+^ originating from oxidative cluster disassembly, as estimated by low- and high-temperature spectra (Fig. S11). Considering that there is minimal degradation during the reductive coupling process (Fig. 4), our findings demonstrate that most of the observed Fe-S cluster degradation during oxidation–reduction occurs during the oxidation process.

## Discussion

Despite multidecade studies on HBx, little is known about the biophysical and biochemical mechanisms by which HBx affects cellular processes during HBV infection. Here we present evidence that HBx is an Fe-S protein and establish the nuclearity and redox properties of the metallocofactor, providing a framework for future studies and development of therapeutics. HBx binds a [2Fe-2S] or [4Fe-4S] cluster depending on the presence or absence of O_2,_ respectively, and we have observed that a small amount of [2Fe-2S] clusters is present in whole *E. coli* cells. The [2Fe-2S] cluster appears to be an oxidative outcome, but its persistence in whole cells also suggests an inability of the *E. coli* biosynthetic machinery to insert intact Fe-S clusters. This compromised Fe-S cluster insertion may be alleviated if HBx is overexpressed in mammalian or insect cells. However, the low solubility of HBx when isolated from insect cells (37) and the variability of HBx overexpression in different mammalian lines pose a significant challenge toward these studies, which nevertheless should be pursued in the near future.

The ability of HBx to coordinate both [2Fe-2S] and [4Fe-4S] clusters is a common feature among sequences from the five HBV genotypes examined (A2, B, C, D, F1), highlighting the potential importance of the Fe-S cofactor in HBx function. HBx is a cysteine-rich polypeptide, offering seven strictly conserved cysteines as likely ligand candidates for the Fe-S cluster. Many of the conserved cysteines have been shown to hold important functional roles, such as C61, C69, and C137, which are crucial for HBx transactivation function (38). We found that single-point cysteine to alanine substitutions allow for wildtype-like Fe-S cluster incorporation and that complete abolition of Fe-S cluster binding is only afforded in a variant lacking all cysteines. Thus, *in vitro* cofactor binding is cysteine dependent but not easily abrogated, suggesting that HBx may reorganize itself to accommodate for Fe-S cluster binding using other available cysteines, protein, or nonprotein residues.

Among the conserved HBx residues, C61, C69, C137, and H139 constitute a CCCH motif reported to coordinate a Zn ion (22). The identified CCCH motif was shown to be critical for HBV replication via degradation of the Smc5/6 complex, but a functional role for Zn binding in this respect has not been identified (22). In our study, expression of DsbC-HBx in media with excess Zn results in a decrease in Fe-S cluster enrichment, reflecting competitive ligation of these two cofactors that may occupy the same binding site. Many Fe-S cluster proteins have historically been misidentified as Zn proteins owing to similar ligand requirements (39). The mitochondrial membrane protein mitoNEET was first annotated as a Zn-finger protein based on primary sequence but was found to instead bind a [2Fe-2S] cluster (39). The catalytic subunit of RNA polymerase (nsp12) from the severe acute respiratory syndrome coronavirus 2 (SARS-cov-2) was first assigned to be Zn dependent based on cryo-EM structures (29). Subsequent studies revealed that nsp12 harbors instead two [4Fe-4S] clusters, one of which is critical for polymerase activity. Zn has been known to inhibit Fe-S biosynthesis *in vivo* by competitively binding to Fe-S scaffold proteins, including IscU and IscA (40). In biochemical studies and during aerobic purification, Zn can supplant an Fe-S cluster that has been oxidatively degraded, leading to the misassignment of proteins as Zn dependent. In some cases, such as with the SARS-cov-2 nsp12, Zn supports limited protein activity by binding to the same site as the Fe-S cluster (29) and partially fulfilling a structural role. Thus, from our present data, we cannot exclude that both Zn and the Fe-S cluster may support HBx cellular activity.

The ability of the Fe-S cluster of HBx to transform from a [2Fe-2S] to a [4Fe-4S] cluster under reducing conditions is especially intriguing, since this is a distinctive behavior observed in Fe-S scaffold proteins. Our spectroscopic studies of the DsbC-HBx [2Fe-2S]→[4Fe-4S] transformation are consistent with a reductive coupling mechanism that was demonstrated for the bacterial IscU and IscA scaffold proteins (24, 35). This mechanism is thought to be the final step in the *de novo* biosynthesis of [4Fe-4S] clusters, allowing scaffold proteins to donate both [2Fe-2S] and [4Fe-4S] clusters to appropriate acceptor proteins (41). Indirect evidence of reductive coupling is also implied in cluster transfer studies of glutaredoxin-type and other A-type scaffold proteins (*e.g.*, GLRX5, SufA, ISCA1/ISCA2) that require reducing agents to transfer clusters to [4Fe-4S] acceptor proteins (42, 43, 44, 45). Reductive coupling in HBx requires the presence of a strong reducing agent, which comes in contrast to A-type scaffolds and glutaredoxins, in which a milder reductant (DTT) can drive the reaction. *A.* *vinelandii isc*Fdx (E_m_ = −344 mV) can also serve as the electron donor for reductive coupling in IscU, but not DsbC-HBx. This observation may not be surprising considering that reduction by ferredoxins is often dependent on protein–protein interactions to facilitate efficient electron transfer (46). The physiological reductant of HBx is presently unknown, but human ferredoxins (*i.e.*, FDX1, FDX2) may serve as better candidates for the reductive coupling in HBx.

There is also some variability in the reversibility of the [2Fe-2S] to [4Fe-4S] conversion among Fe-S scaffold proteins. The majority of [4Fe-4S] clusters in IscU experience O_2_-mediated degradation into mononuclear Fe^3+^, and only a small fraction converts back to [2Fe-2S] clusters (24). In IscA, the process is highly reversible with minimal Fe-S cluster degradation, which can be rationalized considering its proposed role in the maturation of [4Fe-4S] proteins during oxidative stress (35, 47). DsbC-HBx seems to fall in between these two extremes: the Fe-S cluster persists after multiple rounds of oxidation–reduction, albeit with increasing concomitant degradation. Reaction of the reduced Fe-S cluster with O_2_ and concomitant release of Fe may act as sources of ROS via Fe-based Fenton chemistry (48). ROS can cause irreversible oxidative damage by nonspecifically reacting with proteins, lipids, and DNA, leading to carcinogenic mutations (49, 50). There is evidence that HBx is associated with an increase in ROS, thereby causing low-level liver inflammation that promotes carcinogenesis (51, 52). In addition, HBx is proposed to associate with the mitochondria and colocalize with membrane proteins such as cytochrome *c* oxidase, which is a major source of ROS generation in the cell (53, 54). Therefore, partial oxidative degradation of the Fe-S cluster in HBx can offer an alternative route by which HBx can modulate mitochondrial reduction potential or protein activities by ROS formation.

The measured reduction potential of the [4Fe-4S]^2+/1+^ transition in DsbC-HBx is appreciably negative (−520 mV), which may argue against this state’s physiological relevance. The estimated reduction potential of the [4Fe-4S]^2+/1+^ couple in IscU is similarly low at ∼−570 mV (24). The observed low potential of the IscU [4Fe-4S]^2+/1+^ couple may prevent unwanted electron transfer, which may also be true for HBx. However, at present we cannot rule out the possibility that binding of a partner protein or small molecule may cause a shift to a more positive reduction potential. Taken collectively, our results suggest that the [2Fe-2S]^2+^ and [4Fe-4S]^2+^ forms are likely the relevant cofactor states. The redox behavior of the Fe-S cluster in HBx exhibits similarities to Fe-S donating proteins, suggesting that HBx may be involved in Fe regulation and modulate activity of Fe-S containing proteins, such as the iron regulatory protein 1 (IRP1) (55). The cluster sensitivity to molecular oxygen draws parallels to sensor proteins, such as the oxygen-sensing Fe-S containing transcription factor Fumarate nitrate reductase or the redox-sensitive transcriptional activator SoxR (56, 57). The discovery of an Fe-S cluster in HBx adds to a growing body of work identifying Fe-S clusters in viral proteins and opens new exciting directions to provide insight into the mechanisms that lead to carcinogenic outcomes during chronic HBV infection.

## Experimental procedures

### Materials

All chemicals were obtained from Fisher Scientific (unless specified otherwise) and were of high purity grade. NCBI accession codes for HBx genotypes: A2, P69713; B, Q8B8Q5; C, Q9YZS7; D, O11883; F1, Q05499.

### Expression and isolation of DsbC-HBx

The plasmids encoding MBP-HBx or DsbC-HBx were transformed into Rosetta (DE3) competent cells (New England Biolabs) with the pDB1282 plasmid (kindly gifted by Dr Squire Booker, Pennsylvania State University). Cells were grown in standard Lennox Broth (LB) or M9 minimal media for ^57^Fe enrichment at 37 °C with shaking (200 rpm) until an *A*_600_ of ∼0.3 was reached, at which time solid L-Arabinose (0.2% (w/v) final concentration) and solid L-cysteine (200 μM final concentration) were added. At *A*_600_ of ∼0.7, the cells were cold shocked at 4 °C for 1 h. Protein expression was induced by addition of 0.5 mM isopropyl β-d-1-thiogalactopyranoside (IPTG) and supplemented with 250 μM ^56^Fe or 125 μM ^57^Fe. Cells were then incubated at 18 °C for 18 to 20 h and harvested by centrifugation at 7000 rpm for 15 min at 4 °C. Cell pellets were flash frozen in liquid N_2_ and stored at −80 °C prior to purification.

### Aerobic isolation of DsbC-HBx

DsbC-HBx cell pellets were resuspended in Lysis Buffer (50 mM Hepes, 300 mM NaCl, and 10 mM imidazole, 1 mM TCEP, pH 8.0). Phenylmethylsulfonyl fluoride (PMSF) was added to a final concentration of 45 μg/ml. Cells were broken by sonication, and the clarified lysate was applied onto a gravity Ni-NTA column (McLab) equilibrated in Lysis Buffer. The column was washed with Lysis Buffer, followed by an additional washing with Wash Buffer (50 mM Hepes, 300 mM NaCl, 25 mM imidazole, 1 mM TCEP, pH 8.0). The bound protein was eluted with Elution Buffer (50 mM Hepes, 300 mM NaCl, 250 mM imidazole, 1 mM TCEP, pH 8.0), and fractions containing DsbC-HBx were combined and concentrated using a 30 kDa MWCO Amicon Centrifugal Filter (Millipore, Sigma-Aldrich). The concentrated protein was loaded onto a size exclusion S200 column (GE Healthcare), which was equilibrated with Storage Buffer (50 mM Hepes, 150 mM NaCl, 10% glycerol, pH 8.0). Fractions containing the pure protein were pooled and further concentrated.

### Isolation of DsbC-HBx under O_2_-free conditions

DsbC-HBx was purified under strictly O_2_-free conditions in an anaerobic chamber (Coy Labs) using degassed buffer solutions. DsbC-HBx cell pellets were resuspended in Lysis Buffer, followed by addition of 45 μg/ml PMSF, 0.5 mM ^57^Fe, 5 mM sodium dithionite, and 2 mM TCEP. Cells were lysed by sonication for a total time of 30 min (15 s pulse, 59 s pause, 60% amplification), during which Na_2_S was gradually added to a final concentration of 0.5 mM. The clarified lysate was applied onto Ni-NTA column equilibrated in Lysis Buffer. The column was washed with Lysis Buffer, followed by an additional washing with Wash Buffer. The bound protein was eluted with Elution Buffer, and fractions containing DsbC-HBx were combined and concentrated using a 30 kDa MWCO Amicon Centrifugal Filter (Millipore, Sigma-Aldrich). Following the purification steps described above, the obtained DsbC-HBx was stored under anaerobic conditions until further use.

### Isolation of MBP-HBx

MBP-HBx was purified with a MBPtrap column (5 ml, Cytiva) following the same protocols for DsbC-HBx, apart from small changes in buffer composition: Lysis and Wash Buffer (50 mM Hepes, 300 mM NaCl, 1 mM TCEP, pH 8.0), Elution Buffer (50 mM Hepes, 300 mM NaCl, 10 mM Maltose, 1 mM TCEP, pH 8.0).

### Protein and metal quantification

All protein concentrations were determined by the Bradford assay, and protein purity was assessed by SDS-PAGE with Coomassie staining (Thermo Fisher Scientific). Metal analysis of the as-purified proteins was determined by inductively coupled atomic emission spectrometry (ICP-AES). Fe content was determined colorimetrically as described (58).

### Sequence similarity network

The SSN was generated with the web-based Enzyme Function Initiative-Enzyme Similarity Tool (EFI-EST) using the Transactivation protein X InterPro family IPR000236 (13,756 sequences) as an input, which was trimmed to contain only full-length HBx (6792 sequences) (59). For the generation of the SSN, an alignment score of 80 was selected, and the final network was further refined by removing edges with a percent sequence identity that was less than 90.6%.

### Mössbauer spectroscopy

Mössbauer spectra were recorded on a WEB Research spectrometer equipped with a Janis SVT-400 variable-temperature cryostat. The external magnetic field (0.078 T) was applied parallel to the γ-beam. Zero velocity was calibrated as the centroid of the spectrum of α-Fe recorded at room temperature. WMOSS spectral analysis software (www.wmoss.org, WEB Research) was used to simulate and analyze Mössbauer spectra.

### EPR spectroscopy

EPR spectra were recorded on a Bruker E500 Elexsys continuous wave (cw) X-band spectrometer (operating at approximately 9.38 GHz) equipped with a rectangular resonator (TE102) and a continuous-flow cryostat (Oxford 910) with a temperature controller (Oxford ITC 503). All EPR samples were prepared in storage buffer under O_2_-free conditions in an anaerobic glovebox (CoyLab). Samples were reduced with excess sodium dithionite for 30 min at 22 °C prior to being frozen in liquid N_2_, unless stated otherwise. Spin concentration was quantified by double integration of signals using 100 μM CuSO_4_ as a standard.

### Stoichiometric and time-dependent reduction of DsbC-HBx

Stock sodium dithionite solutions were prepared fresh prior to every reaction under strictly anaerobic conditions in Storage Buffer and quantified by measuring the absorbance at 315 nm (ε_315_ = 8043 M^−1^ cm^−1^). For stoichiometric reduction experiments, samples were reacted with sodium dithionite for 30 min at room temperature (∼22 °C) prior to freezing into Mössbauer cups or EPR tubes. For the time-dependent reduction experiments, DsbC-HBx was reacted with excess sodium dithionite (10 mM) at room temperature, and aliquots of the reaction mixture were frozen into Mössbauer cups or EPR tubes at the appropriate timepoints.

### Redox titrations monitored by EPR

DsbC-HBx was first enriched in [4Fe-4S] by reducing the aerobically purified protein with excess sodium dithionite (10 mM), which was further removed by a size exclusion HiLoadTM 16/600 Superdex 200 pg column (Cytiva) housed in the anaerobic chamber. For the titration experiment, 4.4 ml of 972.9 μM protein containing 0.61 Fe/protein was maintained under O_2_-free conditions in the titration cell by continuous flushing with hydrated argon gas. Reduction or oxidation was afforded by dropwise addition of sodium dithionite or potassium ferricyanide, respectively. The potentials were measured with a pH/redox meter (GPHR 1400, Greisinger) using a combination Pt/Ag/AgCl microelectrode (3 M KCl, Mettler Toledo) and are quoted relative to the standard hydrogen electrode. The electrode was calibrated with a saturated quinhydrone solution at pH 8 (1 M Hepes buffer). The following mediators were added and allowed to equilibrate for 15 min with the protein prior to the actual experiment: 50 μM phenazine methosulfate and 100 μM of the following: 2,6-dichlorophenolindophenol, 1,2-napthoquinone, 1,4-napthoquinone, 5-hydroxy-1,4-napthoquinone, methylene blue, 1,4-dihydroxynapthoquinone, indigo carmine, 2-hydroxyl-1,4-napthoquinone, anthraquinone-1,5-disulfonate, phenosafranine, safranine T, methyl viologen, benzyl viologen. The reduction potential was estimated by simulating the fraction of reduced [4Fe-4S]^1+^ for a one-electron reduction process using the Nernst equation.

### Oxygen exposure and redox cycling of DsbC-HBx

For the stoichiometric oxidation experiments, DsbC-HBx:[4Fe-4S] samples were reacted with O_2_ by adding appropriate amounts of O_2_-saturated Storage Buffer (∼1.8 mM O_2_) in a gastight vial for 30 min at room temperature. For time-dependent oxidation, samples were exposed to air at 4 °C before freezing in Mössbauer cups at the respective timepoints. For the redox (reduction–oxidation) cycling, samples were reduced with excess sodium dithionite and exposed to air. Excess dithionite was removed in between redox cycles with Zeba spin desalting columns (ThermoFisher).

## Data availability

All data generated or analyzed during this study are included in this published article (and its supplemental information).

## Supporting information

This article contains supporting information.

## Conflict of interest

The authors declare that they have no conflicts of interest with the contents of this article.
