# Supplementary material for: The HBx protein from hepatitis B virus coordinates a redox-active Fe-S cluster
Source: J Biol Chem. 2022 Feb 8;298(4):101698. doi: 10.1016/j.jbc.2022.101698 (PMC9010755; doi:10.1016/j.jbc.2022.101698)
Supplement: Supplemental Figures S1–S11 and Tables S1–S6 [file mmc1.docx]

Supporting Information for

The HBx protein from Hepatitis B Virus coordinates a redox-active Fe-S cluster

Chie Ueda, Michelle Langton, Jiahua Chen, Maria-Eirini Pandelia*

Department of Biochemistry, Brandeis University, Waltham, Massachusetts, 02453, United States

* Corresponding author

**Email:** [mepandelia@brandeis.edu](mailto:mepandelia@brandeis.edu)

**Contents:**

Supplemental materials and methods

Supplemental figures S1-S11

Supplemental tables S1-S6

Supplemental Materials and Methods

**Preparation of refolded DsbC-HBx and MBP-HBx WT and ΔCys.** ΔCys variants in the DsbC-HBx and MBP-HBx constructs were insoluble and thus purified from lysed cell pellets under denaturing conditions. DsbC-HBx ΔCys cell pellets were resuspended in Pellet Wash Buffer 1 (1% Deoxycholic acid, 1% Triton X-100, 10 M EDTA, pH 8.0) and centrifuged at 4 °C for 30 min at 22,000 x g. Pellets were then washed in 125 mL Pellet Wash Buffer 2 (50 mM HEPES pH 8.0, 0.3 M NaCl, 5 mM ethylenediaminetetraacetic acid (EDTA), 5 mM β-mercaptoethanol, 1% Triton X-100, 3 M Urea), followed by a final wash in Pellet Wash Buffer 3 (50 mM HEPES pH 8.0, 0.3 M NaCl, 5 mM β-mercaptoethanol, 3 M Urea). The washed pellet was then resuspended in Resuspension Buffer 1 (50 mM Tris pH 8.0, 6 M guanidine hydrochloride (GuHCl), 1 mM TCEP) and mixed overnight. Resolubilized protein supernatant was obtained via centrifugation at 4 °C for 30 min at 22,000 x g. Supernatant was loaded on a Ni-NTA column equilibrated in Resuspension Buffer 1 and washed with Resuspension Buffer 1 followed by a wash with 50 mM MES pH 6.0, 6 M GuHCl, 0.5 mM TCEP. Protein was eluted in 20 mM sodium acetate pH 4.5, 8 M Urea, 150 mM NaCl, 0.5 mM TCEP. Eluted protein was degassed and transferred to an anerobic chamber. Fe(NH_4_)_2_(SO_4_)_2_, FeCl_3_, and Na_2_S were added sequentially with 2 min incubation steps in between additions. This step was repeated until the final concentrations of Fe and sulfide were 400 μM each. The sample was then diluted 5-fold in 50 mM HEPES pH 8.0, 300 mM NaCl upon which the solution turned brown in color. The sample was allowed to refold overnight and was further purified using a Ni-NTA column as described above. This procedure was repeated for WT DsbC-HBx as a control.

MBP-HBx ΔCys cell pellets were resuspended in Pellet Wash Buffer 2 and centrifuged at 4 °C for 15 min at 20,000 x g. Pellets were washed an additional two times in Pellet Wash Buffer after which they were resuspended in Resuspension Buffer 2 (5 mM HEPES pH 8.0, 1 mM β-mercaptoethanol, 8 M Urea). The resuspension was centrifuged at 4 °C for 20 min at 20,000 x g, and the supernatant was dialyzed into Lysis Buffer. Protein was further purified using a MBPtrap column as described above. This procedure was repeated for WT MBP-HBx as a control.

Fe-S cluster was reconstituted into the degassed refolded proteins under strictly O_2_-free conditions in an anaerobic chamber. All protein solutions were reduced for 15 min at 4 °C with 10 molar equivalents of dithiothreitol (DTT). Final concentrations of 4 molar equivalents of Fe(NH_4_)_2_(SO_4_)_2_ and 4 molar equivalents of Na_2_S were added to each sample via a total of four and eight additions, respectively, with 15 min incubation steps between each addition. Two aliquots of Fe were added first, followed by four aliquots of sulfide, and this sequence was repeated for each addition. Samples were incubated at 4 °C overnight and desalted via a PD-10 column in Storage Buffer.

**Supplementary Figures and Tables**


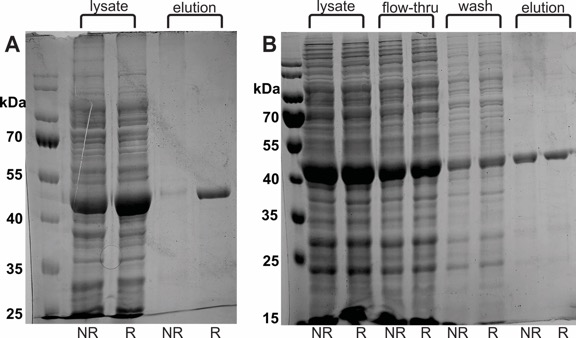


**Fig. S1.** SDS-PAGE gel of *N*-ethylmaleimide (NEM) treated DsbC-HBx purification samples under non-reducing (NR) and reducing (R) conditions. (A) DsbC-HBx samples of lysate and elution collected under aerobic conditions without the presence of TCEP. Disulfide-based aggregation is observed by the absence or decreased intensity of the 42 kDa fusion protein band under non-reducing conditions. (B) DsbC-HBx samples of lysate, flow-through after application to Ni-NTA resin, wash, and elution collected in the presence of 1 mM TCEP. In all samples, the intensity of the DsbC-HBx band is constant with or without reducing agent, indicating the absence of disulfide-based aggregation.


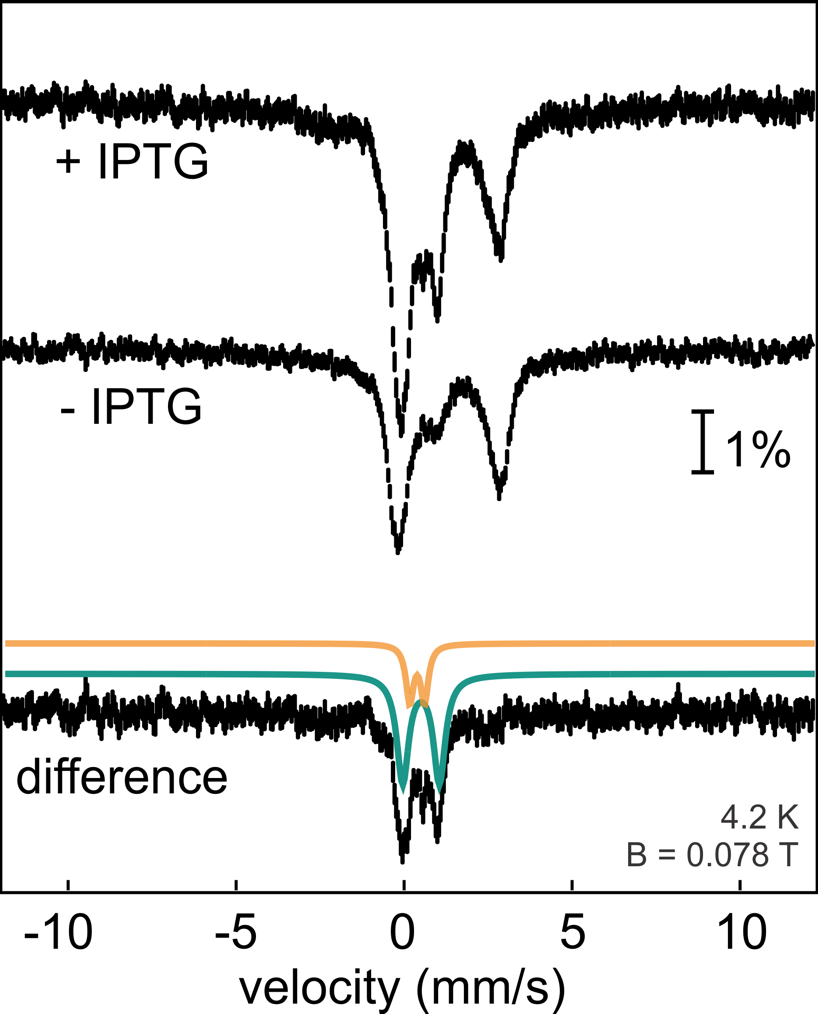


**Fig. S2.** Whole-cell Mössbauer on intact *E. coli* cells. Spectra were recorded at 4.2 K with a magnetic field (B = 78 mT) applied parallel to the γ-beam. T7 (DE3) cells (New England Biolabs, Ipswitch, MA) containing the plasmid for expression of MBP-HBx were grown in M9 minimal media supplemented with 125 µM ^57^Fe. At OD600 ~ 0.7, one cell culture was supplemented with 0.5 mM IPTG to induce protein expression, and both were incubated at 18 °C for 20 h and harvested by centrifugation. Cell pellets were then transferred to Mössbauer cups and frozen in liquid nitrogen. The spectrum of cells without IPTG induction (middle spectrum, -IPTG) was subtracted from the spectrum of cells with IPTG induction (top spectrum, +IPTG), resulting in the difference spectrum (bottom). The difference spectrum shows the presence of both [4Fe-4S] clusters (teal line) and [2Fe-2S] clusters (orange line).


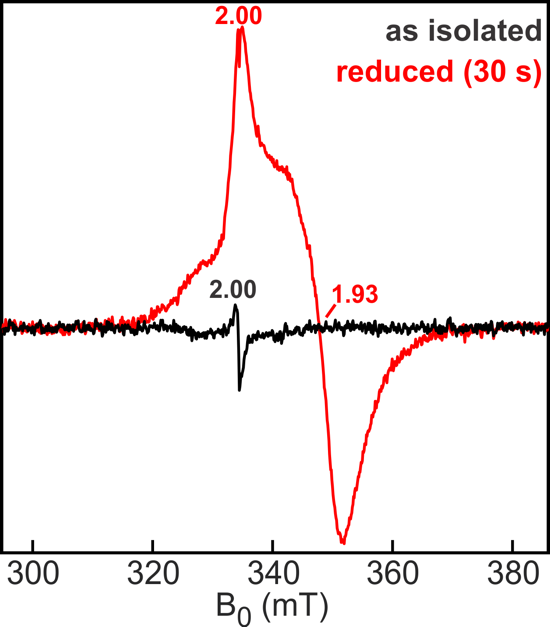


**Fig. S3.** Continuous wave (cw) EPR spectra of DsbC-HBx aerobically isolated in the absence of reducing agent (black line) and reduced with excess sodium dithionite for 30 s (red line). The sharp resonance centered at g ~2.00 in the as-isolated spectrum is not derived from an Fe-S cluster and corresponds to a paramagnetic impurity in the resonator that is not efficiently subtracted.

**
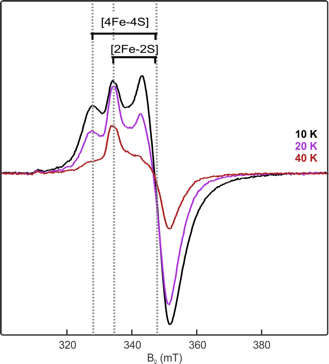
**

**Fig. S4.** Continuous wave (cw) EPR spectra of DsbC-HBx reduced with 1.3 reducing equiv of dithionite per protein for 30 min. The 10 K spectrum (black line) exhibits two sets of signals, the [4Fe-4S]^1+^ and [2Fe-2S]^1+^. As temperature increases, the [4Fe-4S]^1+^ signal decreases due to faster relaxation properties relative to the [2Fe-2S]^1+^cluster signal. At 40 K (red line), we obtain an almost ‘clean’ spectrum of the [2Fe-2S]^1+^ cluster signal. Grey dotted lines indicate the principal g-values corresponding to each type of Fe-S cluster. Experimental conditions: microwave frequency = 9.38 GHz, microwave power = 0.64 mW, modulation amplitude = 1 mT.


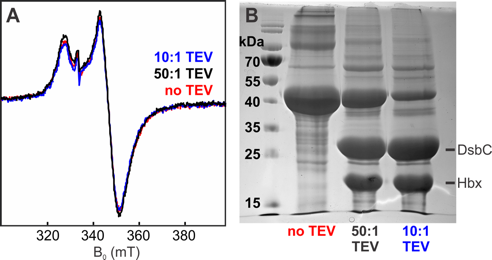


Fig. S5. HBx retains its Fe-S cluster after cleavage of the DsbC tag. (A) cw EPR spectrum of DsbC-HBx without Tobacco Etch Virus (TEV) protease (red line), incubated with 10 molar equivalents of TEV protease (blue line), and with 50 molar equivalents of TEV protease (black line). The signal of the [4Fe-4S]^1+^ is identical in signal intensity for all three samples, indicating no Fe-S cluster loss upon cleavage. TEV protease was incubated with DsbC-HBx for 1 hour at room temperature, then reduced for 30 min with 6 mM sodium dithionite in anaerobic conditions and frozen in EPR tubes. Experimental conditions: temperature = 10 K, microwave frequency = 9.38 GHz, microwave power = 0.2 mW, modulation amplitude = 1 mT. (B) SDS-PAGE gel of the exact EPR samples in (A). The samples containing TEV protease (~27 kDa) demonstrate cleavage of the DsbC-HBx fusion protein by the disappearance of the fusion protein (~42 kDa) and the appearance of the cleaved HBx (~17 kDa).


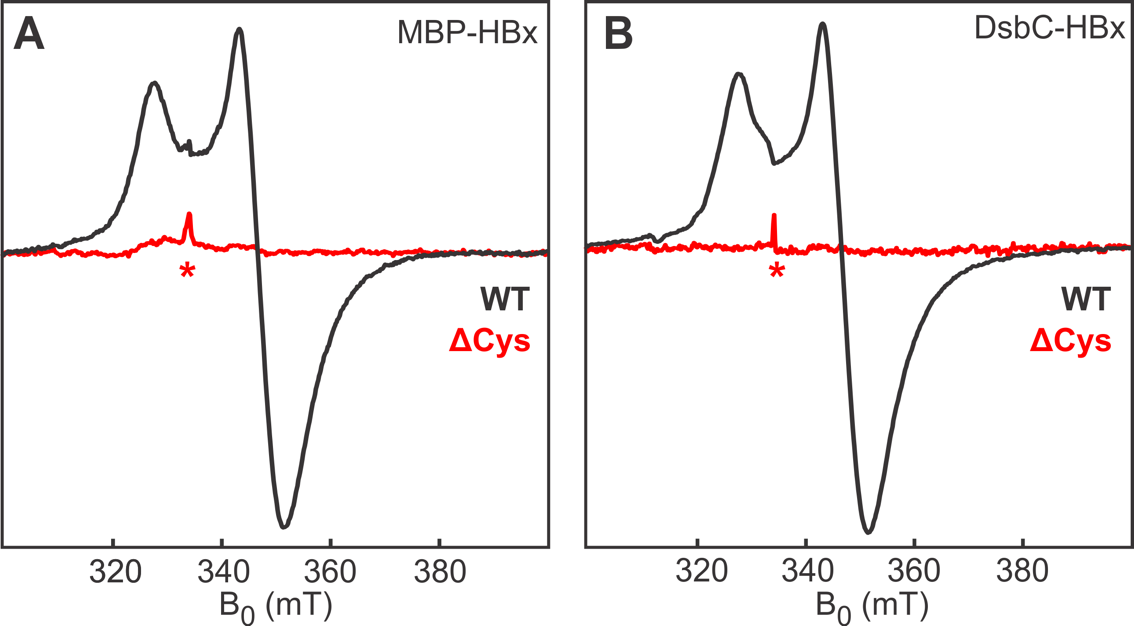


Fig. S6. cw EPR spectra of the wild-type (WT) and all Cys→Ala (ΔCys) HBx fusion proteins. (A) EPR spectrum of WT MBP-HBx (black trace) and ΔCys (red trace). (B) EPR spectrum of WT DsbC-HBx (black trace) and ΔCys (red trace). Experimental conditions: temperature = 10 K, microwave frequency = 9.38 GHz, microwave power = 0.2 mW, modulation amplitude = 1 mT. *The small sharp signal present in all samples is not Fe-S cluster related and comes from a paramagnetic impurity in the resonator cavity.


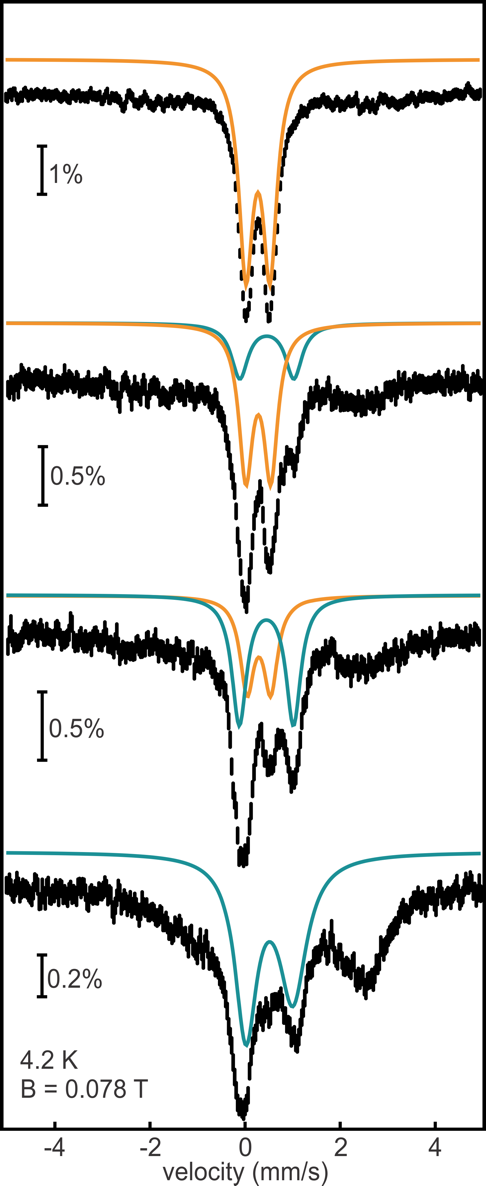


**Fig. S7.** Stoichiometric reduction of aerobically isolated Dsbc-HBx. Mössbauer spectra of DsbC-HBx recorded at 4.2 K with magnetic field (B = 78 mT) applied parallel to the γ-beam. The fits of the quadrupole subspectra corresponding to diamagnetic Fe-S clusters, i.e., [2Fe-2S]^2+^ and [4Fe-4S]^2+^, are represented by orange and teal solid lines, respectively.

**
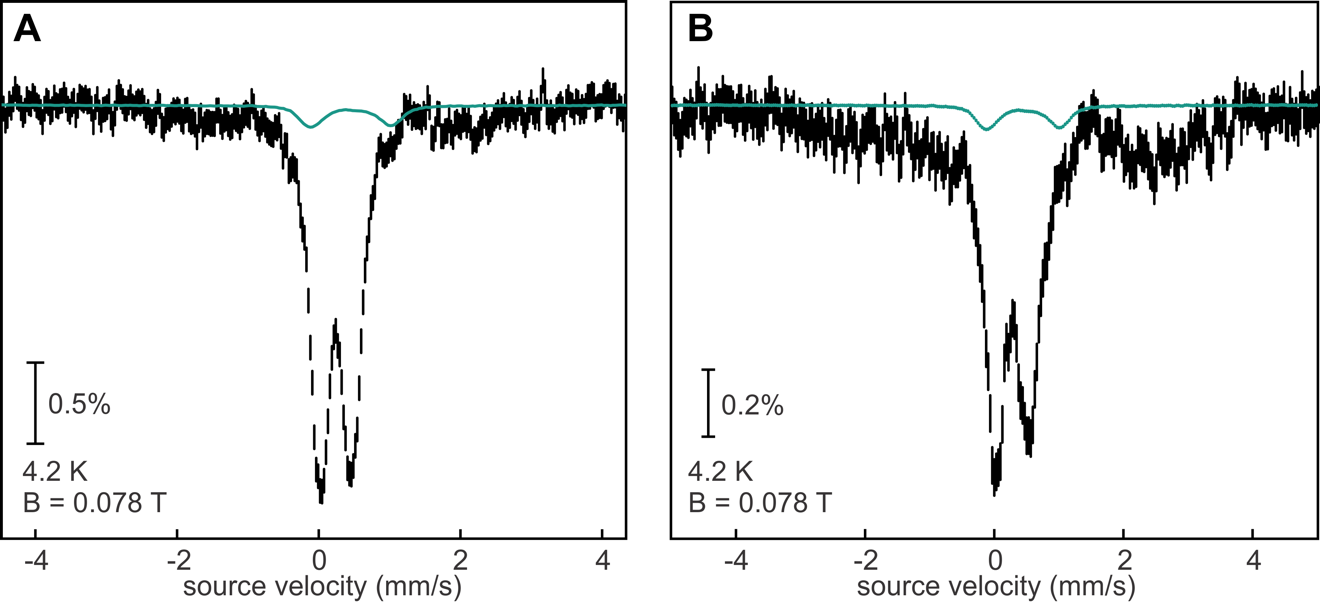
**

**Fig. S8.** Mössbauer spectra of DsbC-HBx recorded at 4.2 K with magnetic field (B = 78 mT) applied parallel to the γ-beam. (A) Aerobically purified DsbC-HBx:[2Fe-2S] incubated with 20 mM DTT for 30 min under anaerobic conditions. The signal (black vertical bars) is dominated by quadrupole doublet with parameters typical of [2Fe-2S]^2+^ clusters (δ = 0.28 mm/s and ΔE_Q_ = 0.51 mm/s, ~82% of spectral intensity). A small shoulder in the signal overlaps with parameters of DsbC:[4Fe-4S] (teal line) contributing at most 5% of the total spectral intensity. (B) Aerobically purified DsbC-HBx:[2Fe-2S] incubated with 1.1 eqv. reduced *isc*Fdx per [2Fe-2S] cluster for 30 min under anaerobic conditions. The spectrum is dominated by a quadrupole doublet with parameters of [2Fe-2S]^2+^ clusters, but there are also significant spectral contributions stemming from mononuclear *S* = 2 Fe^2+^ and broad paramagnetic components (most likely [2Fe-2S]^1+^). A small shoulder in the signal overlaps with parameters of [4Fe-4S]^2+^ (teal line) contributing to at most 5% of the total spectral intensity. We conclude that neither DTT nor *A. vinelandii isc*Fdx drive reductive coupling in DsbC-HBx to a significant effect.


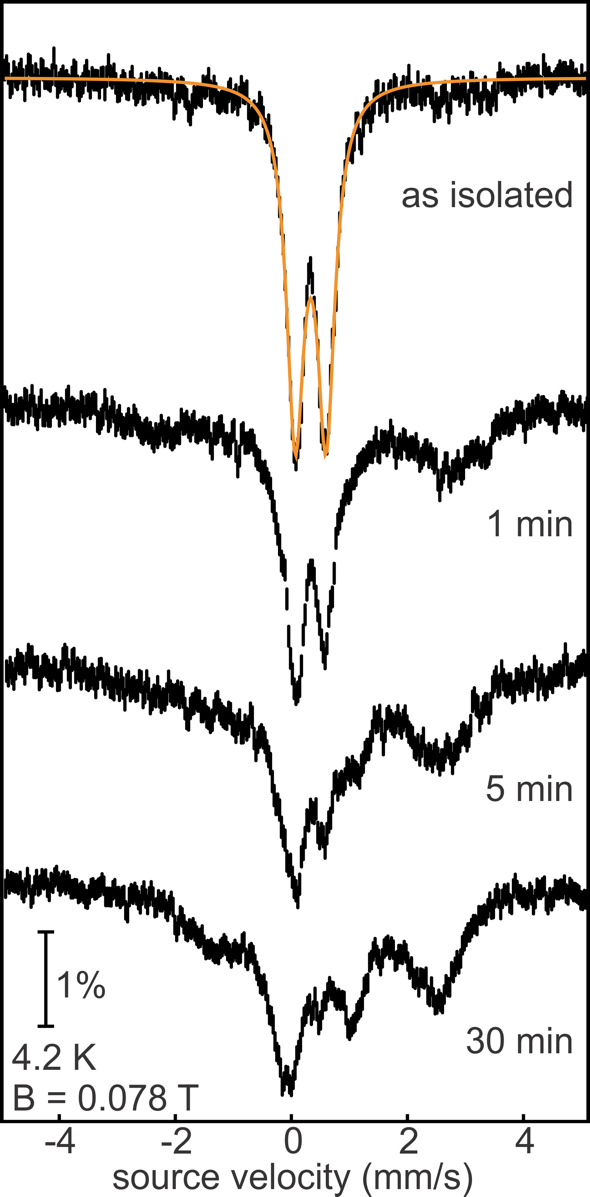


**Fig. S9.** Time-dependent reduction of aerobically isolated Dsbc-HBx. Mössbauer spectra of DsbC-HBx recorded at 4.2 K with a magnetic field (B = 78 mT) applied parallel to the γ-beam. The as-isolated DsbC-HBx (top spectrum) was fitted to a quadrupole doublet with parameters δ = 0.28 mm/s, ΔE_Q_ = 0.51 mm/s maximally amounting to 72% of the signal intensity. Coupled with quantitation of 2.36 Fe/protein, we estimate that there are 0.84 [2Fe-2S]^2+^ per protein bound to DsbC-HBx. The spectra of 1, 5, and 30 min reduced demonstrates the presence of [2Fe-2S]^1+^ and [4Fe-4S]^1+^ as broad components spanning from -2 mm/s to -3 mm/s.


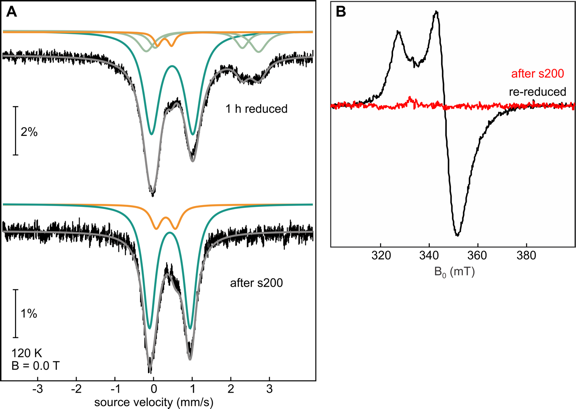


**Fig. S10.** Generation of DsbC-HBx:[4Fe-4S] from aerobically purified DsbC-HBx:[2Fe-2S]. (A) 120 K Mössbauer spectra of DsbC-HBx:[2Fe-2S] reduced with 10 mM NaDT under anaerobic conditions for 1 h (top) and the same sample after a gel-filtration s200 column (bottom). Adventitious Fe^2+^ (light green line, top) has been removed by gel filtration. The total spectrum (gray line) is the sum of the fits of the quadrupole subspectra (teal line, [4Fe-4S]; orange, [2Fe-2S]; light green, Fe^2+^). (B) cw EPR spectra of DsbC-HBx:[2Fe-2S] after reduction and gel-filtration (red line, same sample as A, bottom) and the same sample re-reduced (black line). Reduction of DsbC-HBx:[2Fe-2S] and subsequent gel-filtration results in an EPR silent sample, indicating that the sample is in the [2Fe-2S]^2+^ or [4Fe-4S]^2+^ state. Experimental conditions: temperature = 10 K, microwave frequency = 9.38 GHz, microwave power = 0.2 mW, modulation amplitude = 1 mT.

**
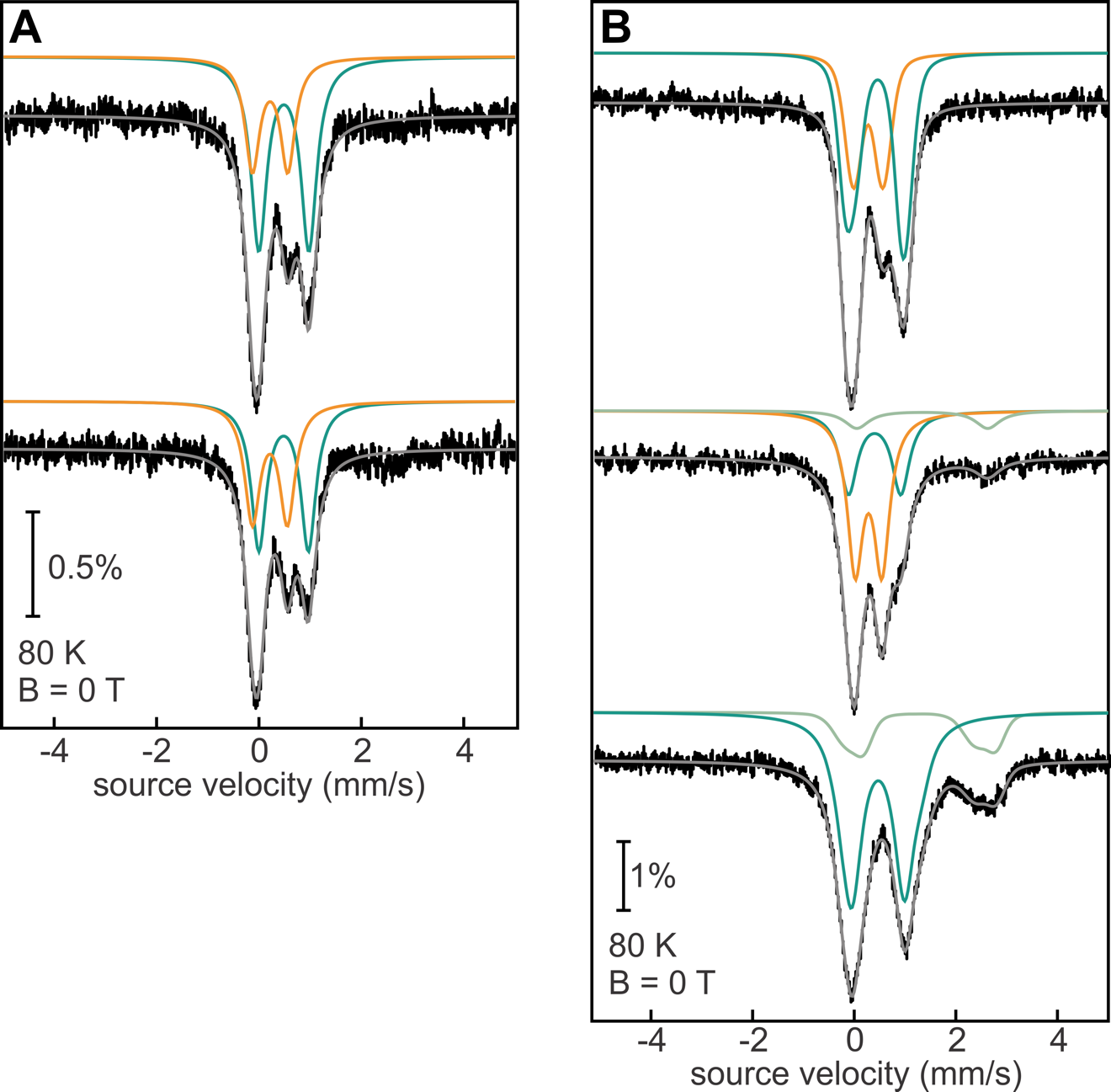
**

**Fig. S11.** Oxygen exposure and redox cycling of DsbC-HBx. Mössbauer spectra were recorded at 80 K in the absence of an external magnetic field. (A) From top to bottom: anaerobically isolated DsbC-HBx exposed to 1 eqv of O_2_, exposed to 2 eqv of O_2_. (B) From top to bottom: DsbC-HBx as-purified anaerobically, exposed to air for 4 h, re-reduced with excess sodium dithionite after 4 h exposure to air. The total simulation (gray lines) for all Mössbauer spectra are sums of the subspectra (orange, [2Fe-2S]; teal, [4Fe-4S]; light green, Fe^2+^).

**Table S1: Metal content of various preparations of HBx fusion proteins**

| **Sample** | **mol Fe per mol protein^a^** |
| --- | --- |
| **MBP-HBx (aerobic isolation)** | 3.4 |
| **MBP-HBx (anaerobic isolation)** | 2.8 |
| **DsbC-HBx (aerobic isolation)** | 1.0 |
| **DsbC-HBx (anaerobic isolation)** | 1.9 |

^a^mol Fe per mol protein was determined by the Ferrozine colorimetric assay

**Table S2: Mössbauer parameters of Fe species**

|  | δ (mm/s)^a^ | ΔE_Q_ (mm/s)^a^ |
| --- | --- | --- |
| [2Fe-2S]^2+^ | 0.28 | 0.50 |
| [2Fe-2S]^1+^ | 0.60, 0.28 | 2.7, 0.51 |
| [4Fe-4S]^2+^ | 0.45 | 1.05 |
| [4Fe-4S]^2+/1+ b^ | 0.55 | 1.3 |
| Fe^2+^ | 1.01-1.32 | 2.3-3.7 |

^a^Average Mössbauer parameters for spectra acquired at 80 K

^b^[4Fe-4S]^1+^ parameters are unable to be deconvoluted from the 2+ state and are cited here as a mixture of 2+/1+ represented by a shift of isomer shift to higher values.

**Table S3: Mössbauer parameters and relative amounts of Fe-S and Fe species in DsbC-HBx from HBV genotypes**

| HBV  genotype | Mössbauer parameters (mm/s)^a^ | [2Fe-2S]^2+^ | [4Fe-4S]^2+^ | Fe^2+^ |
| --- | --- | --- | --- | --- |
| A | δ | - | 0.45 | - |
|  | ΔE_Q_ | - | 1.11 | - |
|  | Relative % | - | 100% | - |
| B | δ | 0.29 | 0.44 | 1.3 |
|  | ΔE_Q_ | 0.58 | 1.16 | 2.6 |
|  | Relative % | 45% | 34% | <10% |
| D | δ | 0.28 | 0.44 | 1.4 |
|  | ΔE_Q_ | 0.51 | 1.14 | 2.6 |
|  | Relative % | 16% | 59% | <5% |
| F1 | δ | 0.3 | 0.44 | 1.3 |
|  | ΔE_Q_ | 0.57 | 1.15 | 2.6 |
|  | Relative % | 13% | 56% | <10% |
| C | δ | 0.29 | 0.44 | 1.3 |
|  | ΔE_Q_ | 0.54 | 1.13 | 2.6 |
|  | Relative % | 36% | 46% | <5% |

^a^Average Mössbauer parameters for spectra acquired at 4.2 K with a magnetic field applied parallel to the γ-beam.

**Table S4: Relative amounts of Fe-S and Fe species quantified in stoichiometric reduction of DsbC-HBx**

|  | [2Fe-2S]^2+/1+^ | | [4Fe-4S]^2+/1+^ | | Fe^2+^ | | Fe^3+^ | |
| --- | --- | --- | --- | --- | --- | --- | --- | --- |
| sample | Relative % | Per protein^a^ | Relative % | Per protein^a^ | Relative % | Per protein^a^ | Relative % | Per protein^a^ |
| As-purified | 84% | 1 | - | - | - | - | 16% | 0.35 |
| 0.7 reducing eqv | 55% | 0.65 | 23% | 0.15 | <5% | - | 15% | 0.35 |
| 1.3 reducing eqv | 31% | 0.35 | 49% | 0.3 | 14% | 0.3 | <5% | - |
| 2.6 reducing eqv | <5% | - | 77% | 0.5 | 18% | 0.4 | - | - |

^a^Fe species per protein was calculated for DsbC-HBx copurified with 2.36 Fe/protein.

**Table S5: Relative amounts of Fe-S and Fe species quantified in time-dependent reduction of DsbC-HBx**

|  | [2Fe-2S]^2+/1+^ | | [4Fe-4S]^2+/1+^ | | Fe^2+^ | | Fe^3+^ | |
| --- | --- | --- | --- | --- | --- | --- | --- | --- |
| sample | Relative % | Per protein^a^ | Relative % | Per protein^a^ | Relative % | Per protein^a^ | Relative % | Per protein^a^ |
| As-purified | 70% | 0.8 | - | - | - | - | 30% | 0.7 |
| 1 min reduced | 35% | 0.4 | 30% | 0.2 | 25% | 0.6 | 10% | 0.2 |
| 5 min reduced | 14% | 0.15 | 57% | 0.3 | 28% | 0.65 | - | - |
| 30 min reduced | - | - | 70% | 0.4 | 30% | 0.7 | - | - |

^a^Fe species per protein was calculated for DsbC-HBx copurified with 2.36 Fe/protein.

**Table S6: Relative amounts of Fe-S and Fe species quantified in redox cycling of DsbC-HBx**

|  |  | [2Fe-2S]^2+/1+^ | | [4Fe-4S]^2+/1+^ | | Fe^2+^ | | Fe^3+^ | |
| --- | --- | --- | --- | --- | --- | --- | --- | --- | --- |
|  | sample | Relative % | Per protein | Relative % | Per protein^a^ | Relative % | Per protein | Relative % | Per protein |
| Fig. 3A^a^ | As-purified | 10% | 0.1 | 90% | 0.5 | - | - | - | - |
|  | 1 eqv O_2_ | 30% | 0.33 | 50% | 0.3 | - | - | 17% | 0.32 |
|  | 2 eqv O_2_ | 40% | 0.4 | 40% | 0.24 | - | - | 20% | 0.44 |
| Fig. 3D^b^ | As-purified | 30% | 0.25 | 70% | 0.32 | - | - | - | - |
|  | Exposed to O_2_ | 38% | 0.35 | 38% | 0.15 | 10% | 0.2 | 10% | 0.2 |
|  | Re-reduced | - | - | 80% | 0.37 | 20% | 0.37 | - | - |

^a^Fe species per protein was calculated based on DsbC-HBx copurified with 2.2 Fe/protein.

^b^Fe species per protein was calculated based on DsbC-HBx copurified with 1.86 Fe/protein.
